# The secondary metabolite hydrogen cyanide protects *Pseudomonas aeruginosa* against sodium hypochlorite-induced oxidative stress

**DOI:** 10.3389/fmicb.2023.1294518

**Published:** 2023-11-16

**Authors:** Waleska Stephanie da Cruz Nizer, Madison Elisabeth Adams, Vasily Inkovskiy, Carole Beaulieu, Joerg Overhage

**Affiliations:** Department of Health Sciences, Carleton University, Ottawa, ON, Canada

**Keywords:** *Pseudomonas aeruginosa*, oxidative stress, sodium hypochlorite, hydrogen cyanide, reactive chlorine species, oxidative stress response, antimicrobial resistance

## Abstract

The high pathogenicity of *Pseudomonas aeruginosa* is attributed to the production of many virulence factors and its resistance to several antimicrobials. Among them, sodium hypochlorite (NaOCl) is a widely used disinfectant due to its strong antimicrobial effect. However, bacteria develop many mechanisms to survive the damage caused by this agent. Therefore, this study aimed to identify novel mechanisms employed by *P. aeruginosa* to resist oxidative stress induced by the strong oxidizing agent NaOCl. We analyzed the growth of the *P. aeruginosa* mutants Δ*katA*, Δ*katE*, Δ*ahpC*, Δ*ahpF*, Δ*msrA* at 1 μg/mL NaOCl, and showed that these known H_2_O_2_ resistance mechanisms are also important for the survival of *P. aeruginosa* under NaOCl stress. We then conducted a screening of the *P. aeruginosa* PA14 transposon insertion mutant library and identified 48 mutants with increased susceptibility toward NaOCl. Among them were 10 mutants with a disrupted *nrdJa, bvlR, hcnA, orn, sucC, cysZ, nuoJ,* PA4166, *opmQ,* or *thiC* gene, which also exhibited a significant growth defect in the presence of NaOCl. We focussed our follow-up experiments (i.e., growth analyzes and kill-kinetics) on mutants with defect in the synthesis of the secondary metabolite hydrogen cyanide (HCN). We showed that HCN produced by *P. aeruginosa* contributes to its resistance toward NaOCl as it acts as a scavenger molecule, quenching the toxic effects of NaOCl.

## 1. Introduction

*Pseudomonas aeruginosa* is an ubiquitous environmental, Gram-negative bacterium and a highly versatile opportunistic human pathogen that can be isolated from soil, water, plants, and animals (Ambreetha and Balachandar, 2022). In humans, it can cause severe and diverse infections of considerable medical importance, such as ventilator-associated pneumonia, endocarditis, urinary tract, and systemic infections, mainly in immunocompromised individuals (Bassetti et al., 2018). One remarkable characteristic of *P. aeruginosa* is its ability to adapt and survive under various and harsh environmental conditions due to a sophisticated network of stress responses, including cold, heat, and oxidative stress responses (Craig et al., 2021; Da Cruz Nizer et al., 2021).

Oxidizing agents are low-molecular-weight compounds that have an increased ability to oxidize other substances by removing electrons and, therefore, are considered potent antimicrobial agents (Finnegan et al., 2010). Among them, reactive oxygen (e.g., hydrogen peroxide, H_2_O_2_) and chlorine (e.g., hypochlorous acid, HOCl) species (ROS and RCS, respectively) are highly reactive molecules produced as by-products of the metabolism of oxygen of living organisms (endogenous production) or encountered by bacterial cells from exogenous sources, such as disinfectants (Bardaweel et al., 2018). In bacterial cells, these molecules oxidize several molecules and disrupt numerous cellular processes. For instance, they react with lipids, proteins, and nucleic acids resulting in membrane damage and affecting protein, DNA, RNA, and energy synthesis (Da Cruz Nizer et al., 2021). Due to their potent activity, ROS and RCS are widely used in many applications as disinfectants in domestic, industrial and hospital settings, water and wastewater treatment, cleaning of wounds, and as antiseptic agents (Da Cruz Nizer et al., 2020, 2021; Gold et al., 2020). Furthermore, H_2_O_2_ and HOCl are also produced by human immune cells as a defense against invading pathogens (Da Cruz Nizer et al., 2020). Overall, HOCl is a more potent oxidizing agent with a much faster antimicrobial effect than H_2_O_2_ (Peskin and Winterbourn, 2001; Winterbourn et al., 2016). It is the active ingredient of sodium hypochlorite (NaOCl; household bleach) and is considered the most commonly used chlorine-based disinfectant and oxidant in drinking water disinfection (Fukuzaki, 2006; Deborde and von Gunten, 2008).

*Pseudomonas aeruginosa* is constantly exposed to oxidative stress, either from endogenous production or exogenous sources, by the use of disinfectants. In this context, this bacterium has developed many mechanisms to survive the toxic effects of these agents. Although HOCl is a stronger oxidant than H_2_O_2_, most research has focused on the H_2_O_2_ responses adopted by *P. aeruginosa,* and only a few studies have identified and characterized specific stress responses and resistance mechanisms against HOCl (Da Cruz Nizer et al., 2020). However, most of these adaptive responses are not specific to a single agent but are part of a general oxidative stress response in *P. aeruginosa*. For instance, detoxifying enzymes, such as catalases, alkyl hydroperoxides, and protein repair systems, such as MrsR, are well-known to be involved in the resistance of *P. aeruginosa* against H_2_O_2_ (Panmanee and Hassett, 2009; Romsang et al., 2013). Furthermore, transcriptional regulators, mainly OxyR, are also crucial for the survival of *P. aeruginosa* under oxidizing conditions (Ochsner et al., 2000). Previous studies have used gene expression analyzes or screening for regulatory proteins to characterize the adaptation of *P. aeruginosa* to HOCl (Groitl et al., 2017; Farrant et al., 2020).

This study aims to identify novel genes involved in the resistance of *P. aeruginosa* to the strong oxidant NaOCl by a targeted screening of genes known to be involved in H_2_O_2_ resistance as well as screening the comprehensive *P. aeruginosa* PA14 mutant library (Liberati et al., 2006) for mutants with increased susceptibility to NaOCl.

## 2. Materials and methods

### 2.1. Bacterial strains, plasmids, and growth conditions

The bacterial strains used in this study included *P. aeruginosa* PA14 wild-type (PA14 WT) (Rahme et al., 1995), *P. aeruginosa* PAO1 WT (Stover et al., 2000) and the entire *P. aeruginosa* PA14 transposon mutant library from Harvard University (Liberati et al., 2006), which was used to screen susceptible mutants. After the selection of PA14 mutants, PAO1 homologs from the PAO1 comprehensive transposon mutant library (Jacobs et al., 2003) were also tested for further verification of our findings. The list of strains used in this study is shown in Supplementary Table S1. Unless otherwise stated, all strains were grown in Lysogeny broth (LB) overnight at 37°C under shaking conditions (220 rpm) and the experiments were conducted using cells in the stationary growth phase. Oxidative stress assays were conducted in BM2 minimal medium [7 mM (NH_4_)_2_SO_4_, 40 mM K_2_HPO_4_, 22 mM KH_2_PO, 0.4% (w/v) glucose, 0.5 mM MgSO_4_, 0.01 mM FeSO_4_, pH 7.0] (Overhage et al., 2008). For easier readability, we used the PAO1 gene numbers for our genes when the gene names were unknown.

The PA2194 PW4739 (Δ*hcnB*) mutant from the PAO1 transposon mutant library (Jacobs et al., 2003) was complemented by the transfer of the *phcnBC* plasmid expressing *hcnBC* (Létoffé et al., 2022), which was kindly provided by Dr. Jean-Marc Ghigo. The complemented mutant Δ*hcnB*-*phcnBC* was grown overnight in LB medium supplemented with 400 μg/mL kanamycin and 2 mM sodium benzoate for inducible gene expression (Létoffé et al., 2022).

### 2.2. Measurement of RCS

NaOCl aqueous solution was used to induce RCS stress. Free chlorine concentration of NaOCl aqueous solutions was determined weekly using DPD Free Chlorine Powder Packs (Thermo Scientific Orion), according to the manufacturer’s instructions. BM2 minimal medium was used to mitigate side reactions between NaOCl and growth medium components. Additionally, to confirm that the addition of NaOCl to BM2 did not reduce the amount of overall RCS, RCS concentration was measured as previously described (Ashby et al., 2020). In this context, addition of 1, 2, and 4 μg/mL NaOCl to BM2 minimal medium did not result in significant reduction in overall RCS levels. In contrast, NaOCl was completely quenched by LB in our control experiment.

### 2.3. Pre-liminary screening for susceptible mutants

To identify genes involved in *P. aeruginosa* stress resistance to NaOCl, we initially screened the *P. aeruginosa* PA14 transposon insertion mutant library (Liberati et al., 2006) for mutants with increased susceptibility to NaOCl. For this, frozen stocks of PA14 mutants arranged in 96-well microtiter plates were transferred to fresh LB medium and incubated for 24 h at 37°C. Then, the mutants were stamped with a 96-pin metal replicator into new 96-well microtiter plates containing NaOCl at 1 μg/mL (½ x MIC) in BM2 growth medium and incubated for 24 h at 37°C. Susceptible mutants (i.e., mutants that did not grow at 1 μg/mL NaOCl) were selected for further analysis.

### 2.4. Minimal inhibitory concentration assay

The MIC for NaOCl and H_2_O_2_ was determined by the standard broth microdilution method in BM2 minimal medium, as described previously (Wiegand et al., 2008). Briefly, *P. aeruginosa* strains were grown overnight in LB at 37°C and 220 rpm. Cells were collected by centrifugation (10,000 rpm for 2 min), washed twice with phosphate-buffered saline (PBS), and resuspended in BM2 medium. The optical density at 600 nm (OD_600nm_) was adjusted to 0.2 (2 × 10^8^ CFU/mL), and 50 μL was mixed in 96-well plates with 50 μL of serial dilutions of NaOCl (0.125–128 μg/mL) or H_2_O_2_ (98–50,000 μg/mL) prepared in BM2 (final cell concentration of 1 × 10^8^ CFU/mL). The plates were incubated for 24 h at 37°C, and the MIC was considered the lowest concentration of oxidizing agent that inhibits the visual growth of bacteria.

### 2.5. Growth curves

*Pseudomonas aeruginosa* overnight cultures grown at 37°C and 220 rpm in LB were washed twice with PBS, resuspended in BM2, and the OD_600nm_ was adjusted to 0.2 (2 × 10^8^ CFU/mL). Then, 50 μL of bacterial suspension and 50 μL of oxidizing agent were mixed in flat-bottom polystyrene 96-well microtiter plates, leading to a final concentration of NaOCl of 1 μg/mL and H_2_O_2_ of 400 μg/mL and the final cell concentration of 1 × 10^8^ CFU/mL. The OD_600nm_ was read every hour for 20 h at 37°C using the Epoch plate reader (Biotek, United States). Growth curves were statistically analyzed by measuring the area under the curve (AUC) using GraphPad Prism version 9.5.1 (San Diego, United States).

### 2.6. Semiquantitative analysis of Hydrogen cyanide (HCN) production

Volatile Hydrogen cyanide production was quantified by the semiquantitative method previously described (Castric, 1977; Létoffé et al., 2022). Briefly, 2 mL of overnight cultures of PAO1 WT, Δ*hcnB*, and Δ*hcnB*-*phcnBC* grown in LB were collected by centrifugation and washed twice with PBS. The cells were resuspended in 2 mL of LB and transferred to a small Petri dish (35 cm diameter) placed in the middle of a 100-mm diameter Petri dish. The small petri dish was covered with chromatography paper soaked in HCN detection reagent: 100 mg of copper (II) ethyl acetoacetate and 100 mg of 4,4′-methylenebis-(*N,N*-dimethylaniline) solubilized in 20 mL chloroform (Létoffé et al., 2022). The large petri dish was then closed and incubated at 37°C for 24 h under static conditions. HCN production was detected by blue color formation on the chromatography paper. Two mM sodium benzoate was added to Δ*hcnB*-*phcnBC* cells.

### 2.7. Time-kill kinetics experiments

*Pseudomonas aeruginosa* was grown overnight in LB at 37°C under shaking conditions, collected by centrifugation, washed twice, and resuspended in PBS. The OD_600nm_ was adjusted to 0.1, and the bacterial suspensions were treated with NaOCl at 2 μg/mL for 5, 15, 30, and 60 min. For the experiments using the supernatant, PAO1 WT, Δ*hcnB* and Δ*hcnB*-*phcnBC* were grown in BM2 overnight. Then, PAO1 WT, Δ*hcnB*, or Δ*hcnB*-*phcnBC* culture supernatants were collected by centrifugation followed by sterile filtration using a 0.22 μm filter. On the other hand, Δ*hcnB* cells were collected by centrifugation, washed twice with PBS, and the OD_600nm_ was adjusted to 0.1 by diluting the cells in PAO1 WT, Δ*hcnB*, or Δ*hcnB*-*phcnBC* supernatants. The bacterial suspensions were treated with 4 μg/mL NaOCl for 60 min.

After the treatments, 10 mM Na_2_S_2_O_3_ was added to the samples to quench NaOCl, and the cells were serially diluted and plated out on LB agar plates using the drop plate method previously described (Herigstad et al., 2001).

### 2.8. Statistical analysis

Statistical analyzes were performed using GraphPad Prism software version 9.5.1 (San Diego, United States). The Shapiro–Wilk test was used to confirm the normality of the data. Parametric data were analyzed by One Way ANOVA, followed by Tukey or Dunnett’s post-test for multiple comparisons or Student’s t-test for comparison between two groups. Non-parametric data were analyzed by the t-test and Mann–Whitney test for comparison between two groups. All experiments were performed in at least three independent experiments, and results were considered statistically significant when *p* < 0.05.

## 3. Results

### 3.1. H_2_O_2_ Detoxifying mechanisms also contribute to the NaOCl survival of *Pseudomonas aeruginosa*

Previous work on H_2_O_2_ has identified several detoxifying enzymes and oxidative stress repair systems in *P. aeruginosa*. To evaluate if these previously described genes involved in H_2_O_2_ adaptation also play a role in the adaptation of *P. aeruginosa* to NaOCl, we examined growth of the *P. aeruginosa* PAO1 and PA14 mutants Δ*katA* and Δ*katE* (catalases), Δ*ahpC* and Δ*ahpF* (alkyl hydroperoxide reductase), Δ*msrA* (methionine sulfoxide reductase), and Δ*ohrR* (organic hydroperoxide resistance protein) exposed to NaOCl at 1 μg/mL for 20 h at 37°C. This sub-lethal concentration was chosen based on the MIC of the WT strains (2 μg/mL). In accordance with Farrant et al. (2020), mutants were considered to possess a susceptibility phenotype when they presented an increased lag phase of >3 h compared to the WT strain. Furthermore, to statistically analyze the growth curves obtained, we measured the AUC. AUC, also known as growth potential (Todor et al., 2014), is a metric to quantify the cumulative effect of overall growth over time (Sprouffske and Wagner, 2016).

**Figure 1:**
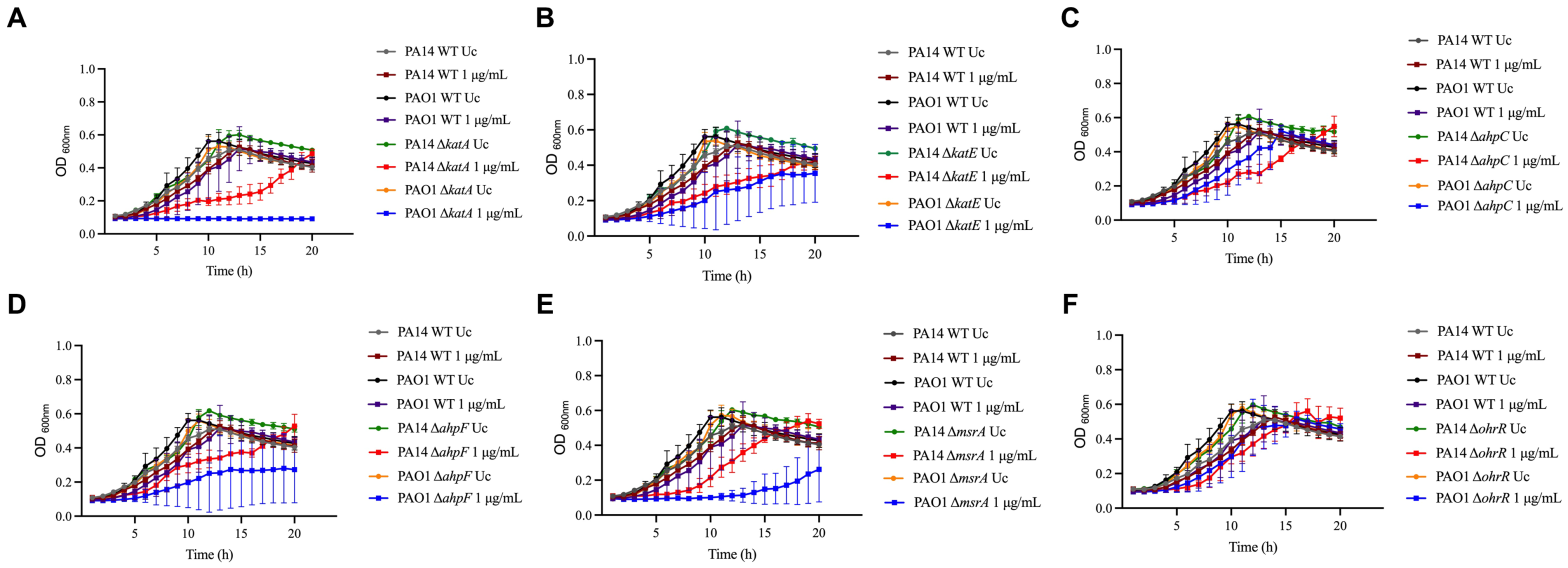
Susceptibility of known-H_2_O_2_ resistance mechanisms to NaOCl. Overnight cultures were grown in LB at 37°C and 220 rpm. Then, cells were washed twice with PBS, resuspended in BM2 minimal medium, and treated with NaOCl at 1 μg/mL (final cell concentration of 1 × 10^8^ CFU/mL) for 20 h at 37°C. OD_600nm_ was recorded every hour using an epoch plate reader. **(A)** Δ*katA*; **(B)** Δ*katE*; **(C)** Δ*ahpC*; **(D)** Δ*ahpF*; **(E)** Δ*msrA*; **(F)** Δ*ohrR*. WT: wild-type; Uc, untreated control.

*Pseudomonas aeruginosa* PAO1 and PA14 WT treated with 1 μg/mL NaOCl took approximately 5–6 h to reach an OD_600nm_ of 0.2 (double the initial OD). Overall, all mutants presented reduced growth at 1 μg/mL NaOCl compared to the WT strains. Furthermore, the AUCs were statistically significant for both the PA14 and PAO1 mutant strains compared to the WT strains treated with 1 μg/mL NaOCl. An OhrR mutant, a transcriptional repressor involved in oxidative stress response in *P. aeruginosa*, was used as a control and presented growth compared to the untreated controls and WT strains (Figure 1; Table 1). Of note, the PA14 Δ*katA* mutant (Figure 1A) required 11 h to reach an OD_600nm_ of 0.2, nearly twice as long as the time needed for the PA14 WT strain, and the PAO1 mutant did not show any growth. Furthermore, the Δ*katA* PA14 mutant exhibited an AUC approximately 2x smaller than that of the PA14 WT strain, whereas the Δ*katA* PAO1 mutant had an AUC more than 4x smaller than the PAO1 WT strain.

**Table 1:** MIC and area under the curve (AUC) of growth curves of *Pseudomonas aeruginosa* strains exposed to 1 μg/mL NaOCl.

*P. aeruginosa* strains		MIC (μg/mL)	Area under the curve (AUC)	
			Untreated Control	1 μg/mL NaOCl
PAO1 WT		2	9.288 ± 0.875	7.946 ± 0.874
PA14 WT		2	8.115 ± 0.983	7.310 ± 0.912
*ΔkatA*	PA4236	1	6.823 ± 0.012	1.761 ± 0.054*
	PA14_09150	1	7.767 ± 0.097	4.269 ± 0.545*
*ΔkatE*	PA2147	1	6.797 ± 0.137	3.823 ± 1.790*
	PA14_36810	1	7.687 ± 0.049	4.093 ± 0.989*
*ΔahpC*	PA0139	1	6.965 ± 0.045	5.417 ± 1.017*
	PA14_01710	1	7.616 ± 0.184	5.002 ± 0.313*
*ΔahpF*	PA0140	1	6.924 ± 0.023	3.812 ± 0.645*
	PA14_01720	1	7.704 ± 0.203	5.410 ± 0.369*
*ΔmsrA*	PA5018	1	7.133 ± 0.092	2.394 ± 0.883*
	PA14_66330	1	7.651 ± 0.207	5.346 ± 0.520*
*ΔohrR*	PA2849	4	7.198 ± 0.154	6.106 ± 0.877
	PA14_27230	4	7.455 ± 0.188	5.871 ± 0.783

WT, wild-type. Data were analyzed by One-Way ANOVA and compared with the PA14 or PAO1 WT strains treated with 1 μg/mL NaOCl. *p < 0.05. AUC represents the average ± standard deviation of at least three independent experiments.

In addition to the increased susceptibility in the growth analyzes, these mutants presented a 2-fold increase in susceptibility in MIC testing (MIC of 1 μg/mL; ½ x MIC of the WT), except for OhrR, which presented a MIC of 4 μg/mL (Table 1). These results demonstrate the importance of these repair systems in detoxifying toxic oxygen species, including NaOCl.

### 3.2. PA14 transposon mutant library screening for identification of novel genes involved in NaOCl resistance

**Figure 2:**
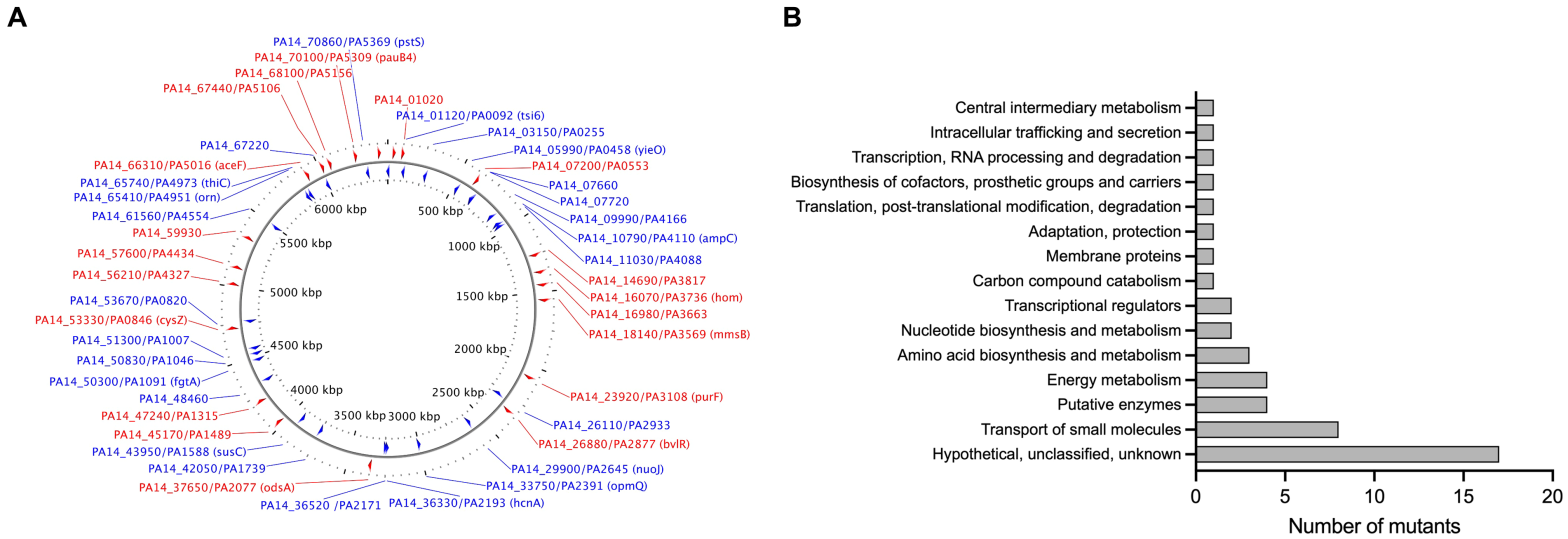
**(A)** Map of the *P. aeruginosa* genome with genes identified in the MIC screening. The image was generated by CGview (Stothard and Wishart, 2005). Red: Forward genes; Blue, Reverse genes. **(B)** Functional classification of genes identified in the MIC screening based on the *P. aeruginosa* genome classification.

To identify novel genes involved in NaOCl resistance, we screened the comprehensive Harvard PA14 transposon insertion mutant library (Liberati et al., 2006) for mutants with increased susceptibility to NaOCl. In the preliminary screening, we exposed the PA14 mutants to NaOCl at 1 μg/mL in BM2 minimal growth medium. Mutants not showing visual growth at this concentration were selected for further MIC testing to confirm their phenotypes. In total, 48 PA14 mutants with MIC of 0.5 and 1 μg/mL (¼ and ½ x MIC of PA14 WT) were identified and selected for further analysis (Figure 2A; Supplementary Table S2). Most of the mutants identified in this preliminary screening have a mutation in genes with unknown function (17/48), followed by genes involved in the transport of small molecules (8/48), such as ABC transporter, sulfate uptake protein, and major facilitator superfamily (MFS) transporter (Figure 2B). Furthermore, among the 48 mutants identified, 9 presented MIC values of 0.5 μg/mL [PA2077 (hypothetical protein), PA2193 (Δ*hcnA*, cyanide production), PA0846 (sulfate uptake protein), PA4110 (Δ*ampC*, cephalosporinase), PA5446 (hypothetical protein), PA0040 (hemolysin activation/secretion protein), PA1046 (hypothetical protein), PA4973 (Δ*thiC*, thiamin biosynthesis protein ThiC), and PA1315 (transcriptional regulator)].

### 3.3. Growth kinetic analyzes of mutants identified in the planktonic screening

**Figure 3:**
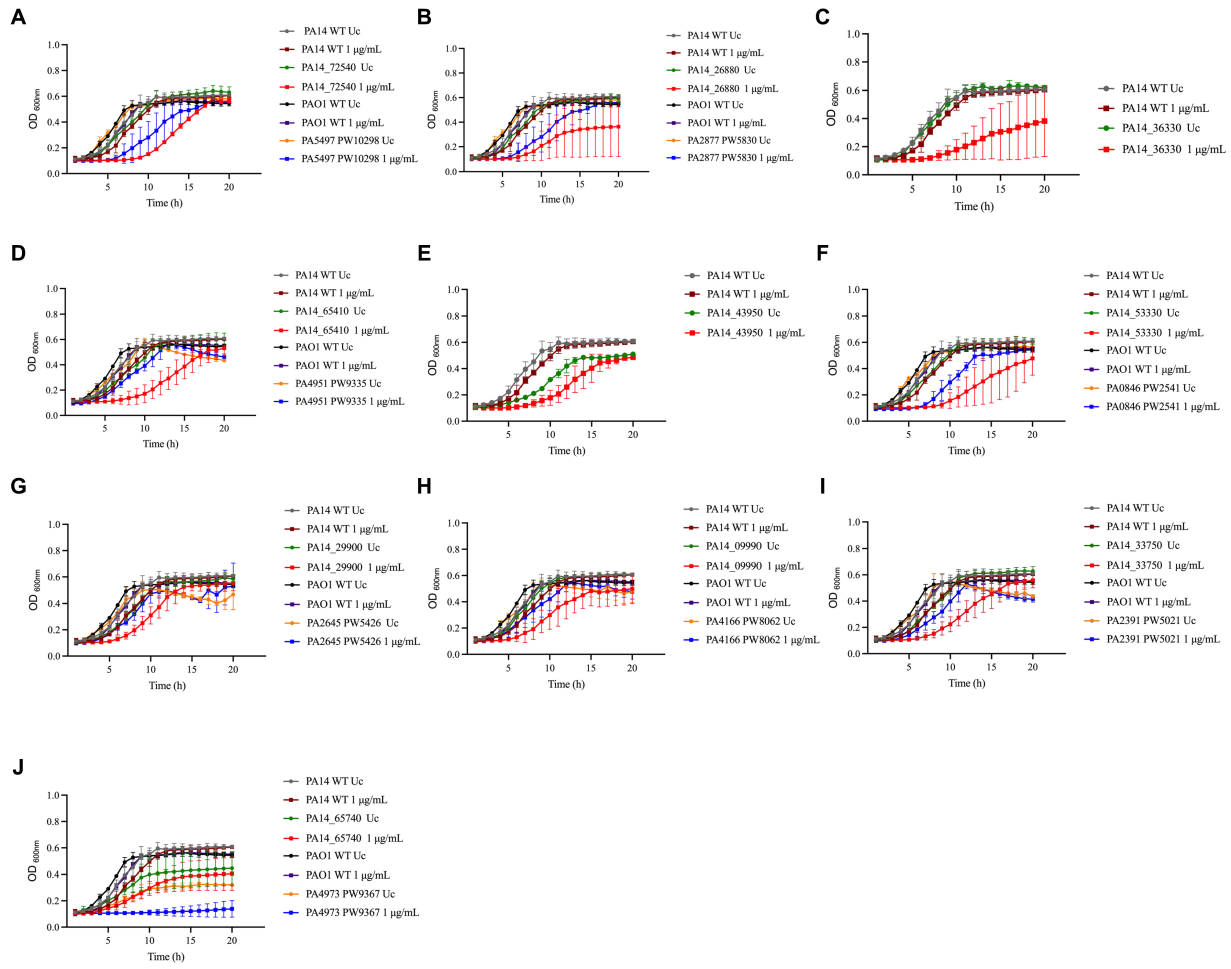
Susceptibility of PA14 mutants and PAO1 homologs identified in the screening to NaOCl. Overnight cultures were grown in LB at 37°C and 220 rpm. Then, cells were washed twice, resuspended in BM2 minimal medium, and treated with NaOCl at 1 μg/mL (final cell concentration of 1 × 10^8^ CFU/mL) for 20 h at 37°C. OD_600nm_ was recorded every hour using an epoch plate reader. **(A)** Δ*nrdJa* (PA14_72540/PA5497); **(B)** Δ*bvlR* (PA14_26880/PA2877); **(C)** Δ*hcnA* (PA14_36330); **(D)** Δ*orn* (PA14_65410/PA4951); **(E)** Δ*sucC* (PA14_43950); **(F)** Δ*cysZ* (PA14_53330/PA0846); **(G)** Δ*nuoJ* (PA14_29900/PA2645); **(H)** PA14_09990/PA4166; **(I)** Δ*opmQ* (PA14_33750/PA2391); **(J)** Δ*thiC* (PA14_65740/PA4973). WT: wild-type; Uc: untreated control.

To further characterize the susceptibility phenotype of the mutants identified in the MIC screening in more detail, we analyzed the growth of these 48 mutants in the presence of 1 μg/mL NaOCl for 20 h at 37°C in microtiter plates. Overall, 10 PA14 mutants identified in the library screening also presented a significant delay in growth in the presence of NaOCl compared to the WT-treated strains (i.e., more than 10 h to double the initial OD). Table 2 shows the 10 mutants identified in the PA14 mutant library screening, and Figure 3 illustrates their growth kinetics at 1 μg/mL NaOCl. Most of the mutants reached the OD_600nm_ of 0.2 (double the initial cell concentration) after 11 h of incubation, while it took approximately 5–6 h for the WT strains to get to this OD. Among them, Δ*bvlR*, Δ*hcnA,* and Δ*thiC* presented overall reduced growth at 1 μg/mL NaOCl, reaching the maximum OD_600nm_ of 0.364, 0.382, and 0.405, respectively, after 20 h compared to the PA14 WT (OD_600nm_ of 0.6 after 20 h).

**Table 2:** Susceptibility to *Pseudomonas aeruginosa* mutants to NaOCl.

Locus name	PAO1 homolog	Gene name	Gene description	Functional category	MIC (μg/mL)
PA14_72540	PA5497	*nrdJa*	Putative ribonucleotide reductase	Nucleotide biosynthesis and metabolism	1
PA14_26880	PA2877	*bvlR*	Putative transcriptional regulator, LysR family	Transcriptional regulators	1
PA14_36330	PA2193*	*hcnA*	Hydrogen cyanide synthase	Central intermediary metabolism	0.5
PA14_65410	PA4951	*orn*	Oligoribonuclease	Transcription, RNA processing, and degradation	1
PA14_43950	PA1588*	*sucC*	Succinyl-CoA synthetase beta subunit	Energy metabolism	1
PA14_53330	PA0846	*cysZ*	Probable sulfate uptake protein	Transport of small molecules	0.5
PA14_29900	PA2645	*nuoJ*	NADH dehydrogenase I chain J	Energy metabolism	1
PA14_09990	PA4166		Putative acetyltransferase	Putative enzymes	1
PA14_33750	PA2391	*opmQ*	Putative outer membrane protein precursor	Transport of small molecules	1
PA14_65740	PA4973	*thiC*	Thiamin biosynthesis protein ThiC	Biosynthesis of cofactors, prosthetic groups, and carriers	0.5
PA14 WT					2
PA01 WT					2

*PAO1 homolog unavailable for testing.

To evaluate if our findings are strain-specific, we also assessed the growth of the PAO1 mutant homologs from the PAO1 two-allele transposon mutant library from the University of Washington Genome Center (Jacobs et al., 2003) under the same experimental conditions over time (Figure 3). Moreover, to statistically analyze the growth curves obtained for the PA14 and PAO1 mutants, we calculated the AUC and compared the values obtained with the AUC of 1 μg/mL NaOCl-treated WT strains (Table 3). The mutants Δ*nrdJa*, Δ*cysZ*, Δ*opmQ*, and Δ*thiC* presented delayed growth (i.e., lag phase >3 h than the WT strains) and statistically different AUCs compared to the NaOCl-treated WT strains for both PA14 and PAO1 mutants, suggesting that the phenotype found is not strain specific. PAO1 homologs for the mutants Δ*hcnA* and Δ*sucC* were unavailable for testing.

**Table 3:** Area under the curve (AUC) of NaOCl and H_2_O_2_ growth curves of NaOCl-susceptible *Pseudomonas aeruginosa* strains identified in the screening.

Gene name	*P. aeruginosa* strains	NaOCl		H_2_O_2_	
		Untreated	1 μg/mL	Untreated	400 μg/mL
	**PAO1 WT**	9.288 ± 0.875	7.946 ± 0.874	9.385 ± 0.747	5.542 ± 0.775
	**PA14 WT**	8.115 ± 0.983	7.310 ± 0.912	8.279 ± 0.748	4.253 ± 0.956
*nrdJa*	PA5497 PW10298	8.776 ± 0.056	6.058 ± 1.027*	8.626 ± 0.195	4.264 ± 0.416
	PA14_72540	8.572 ± 0.525	5.031 ± 0.122*	7.555 ± 0.149	4.644 ± 1.003
*bvlR*	PA2877 PW10298	8.776 ± 0.056	6.877± 0.665	8.846 ± 0.254	6.494 ± 0.68
	PA14_26880	8.096 ± 0.110	4.295 ± 2.310*	7.506 ± 0.115	4.872 ± 1.934
*hcnA*	PA14_36330	8.695 ± 0.365	4.034 ± 1.968*	8.178 ± 0.192	3.863 ± 0.674
*orn*	PA4951 PW9335	7.635 ± 0.308	6.868 ± 0.372	9.055 ± 0.553	4.645 ± 0.518
	PA14_65410	7.991 ± 0.327	4.880 ± 1.049*	7.502 ± 0.031	3.828 ± 0.452
*sucC*	PA14_43950	6.094 ± 0.136	4.787 ± 0.747*	6.927 ± 0.181	3.254 ± 0.509
*cysZ*	PA0846 PW2541	8.520 ± 0.068	5.991 ± 0.442*	8.385 ± 0.152	5.324 ± 0.150
	PA14_53330	8.229 ± 0.283	4.302 ± 1.661*	7.563 ± 0.144	4.216 ± 0.679
*nuoJ*	PA2645 PW5426	7.327 ± 0.543	6.765 ± 0.840	7.619 ± 0.212	5.524 ± 0.564
	PA14_29900	7.851 ± 0.119	6.328 ± 0.503	6.146 ± 0.214	3.429 ± 1.247
	PA4166 PW8062	7.812 ± 0.670	7.102 ± 0.814	8.552 ± 0.116	4.919 ± 0.609
	PA14_09990	8.414 ± 0.260	5.794 ± 1.315	7.793 ± 0.159	4.171 ± 0.928
*opmQ*	PA2391 PW5021	7.711 ± 0.348	6.339 ± 0.236*	8.639 ± 0.048	4.529 ± 0.775
	PA14_33750	8.222 ± 0.337	5.525 ± 0.978*	7.657 ± 0.030	4.226 ± 0.051
*thiC*	PA4973 PW9367	4.765 ± 0.356	2.194 ± 0.354*	4.609 ± 0.241	1.864 ± 0.015*
	PA14_65740	6.383 ± 1.677	5.272 ± 1.088*	4.974 ± 0.381	1.891 ± 0.098*

WT, wild-type. Data were analyzed by One-Way ANOVA and compared with the PA14 or PAO1 WT strains treated with 1 μg/mL NaOCl. *p < 0.05. AUC represents the average ± standard deviation of at least three independent experiments.

**Figure 4:**
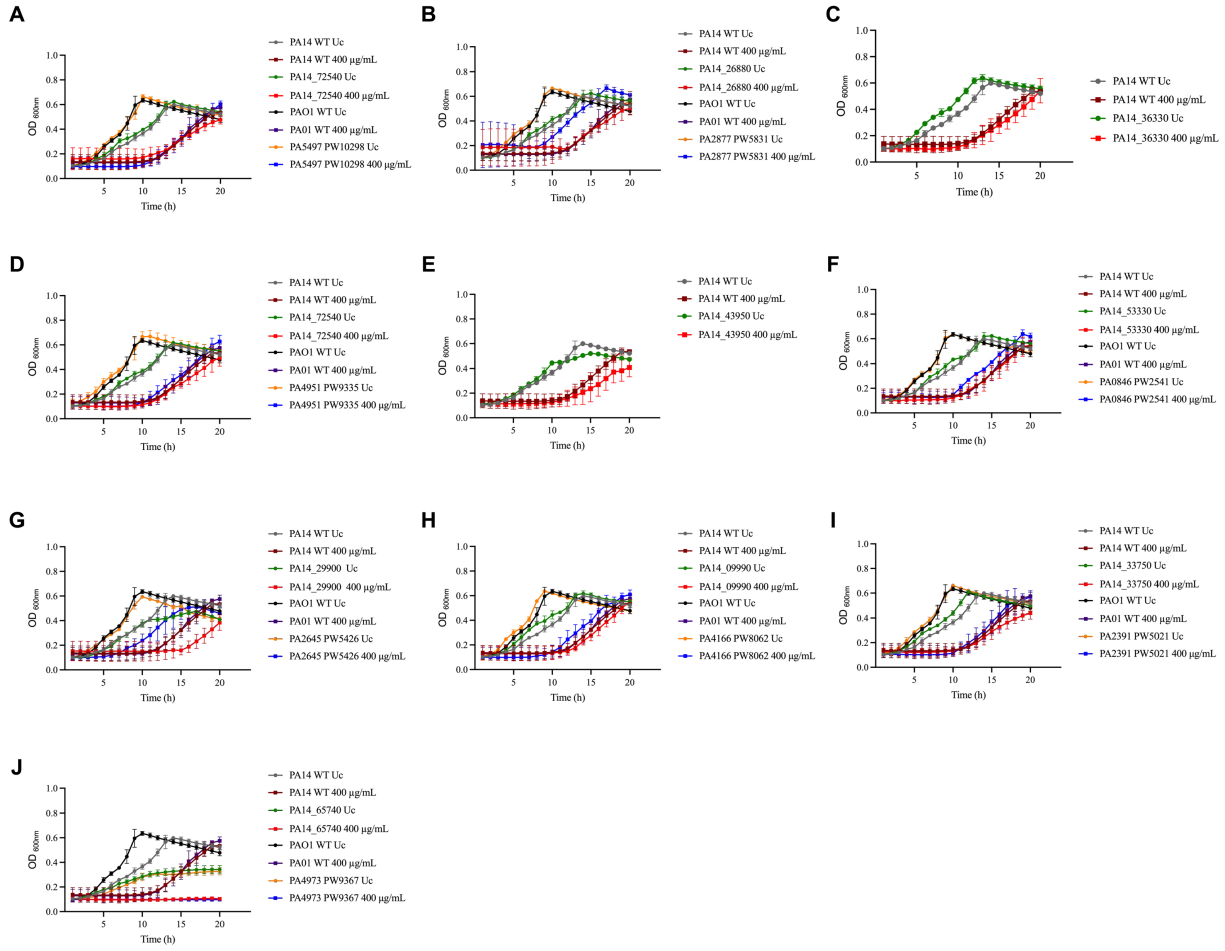
Susceptibility of PA14 mutants and PAO1 homologs identified in the screening to H_2_O_2_. Overnight cultures were grown in LB at 37°C and 220 rpm. Then, cells were washed twice, resuspended in BM2 minimal medium, and treated with H_2_O_2_ at 400 μg/mL (final cell concentration of 1 × 10^8^ CFU/mL) for 20 h at 37°C. OD_600nm_ was recorded every hour using an epoch plate reader. **(A)** Δ*nrdJa* (PA14_72540/PA5497); **(B)** Δ*bvlR* (PA14_26880/PA2877); **(C)** Δ*hcnA* (PA14_36330); **(D)** Δ*orn* (PA14_65410/PA4951); **(E)** Δ*sucC* (PA14_43950); **(F)** Δ*cysZ* (PA14_53330/PA0846); **(G)** Δ*nuoJ* (PA14_29900/PA2645); **(H)** PA14_09990/PA4166; **(I)** Δ*opmQ* (PA14_33750/PA2391); **(J)** Δ*thiC* (PA14_65740/PA4973). WT: wild-type; Uc: untreated control.

Most of the NaOCl responses previously reported in other studies are not specific to NaOCl but are rather employed by bacteria to survive the stress caused by different oxidizing agents (Da Cruz Nizer et al., 2021). Therefore, we conducted growth kinetics over time of the PA14 mutants identified and their PAO1 homologs to investigate if these genes are NaOCl-specific by exposing the mutants to 400 μg/mL H_2_O_2_. This concentration was chosen based on the growth curve of PA14 and PAO1 WT strains previously conducted (Supplementary Figure S1). As shown in the growth analyzes of Figure 4 and the AUC values in Table 3, the PA14 and PAO1 homologs tested were not susceptible to H_2_O_2_ under our experimental conditions, except for Δ*thiC*, which did not grow in the presence of 400 μg/mL H_2_O_2_. These results indicate that the susceptibility phenotypes found in our experiments are rather specific to NaOCl under our experimental conditions.

### 3.4. HCN affects NaOCl resistance in *Pseudomonas aeruginosa*

Among the mutants identified in our screening and follow-up MIC and growth analyzes (Table 2) was the Δ*hcnA* mutant, which presented increased susceptibility to NaOCl. For instance, it took approximately 11 h for the Δ*hcnA* mutant to reach an OD_600nm_ of 0.2, while the PA14 WT strain grew to an OD_600nm_ of 0.2 in only 5–6 h. After 20 h of growth, the Δ*hcnA* mutant exhibited an OD_600nm_ of 0.382, while the PA14 WT showed an OD_600nm_ of 0.6. Furthermore, the Δ*hcnA* mutant presented a MIC of 0.5 μg/mL, which was ¼ x MIC of PA14 WT. Given this pronounced increase in susceptibility, we focused the following analyzes on HCN and its contribution to NaOCl resistance.

**Figure 5:**
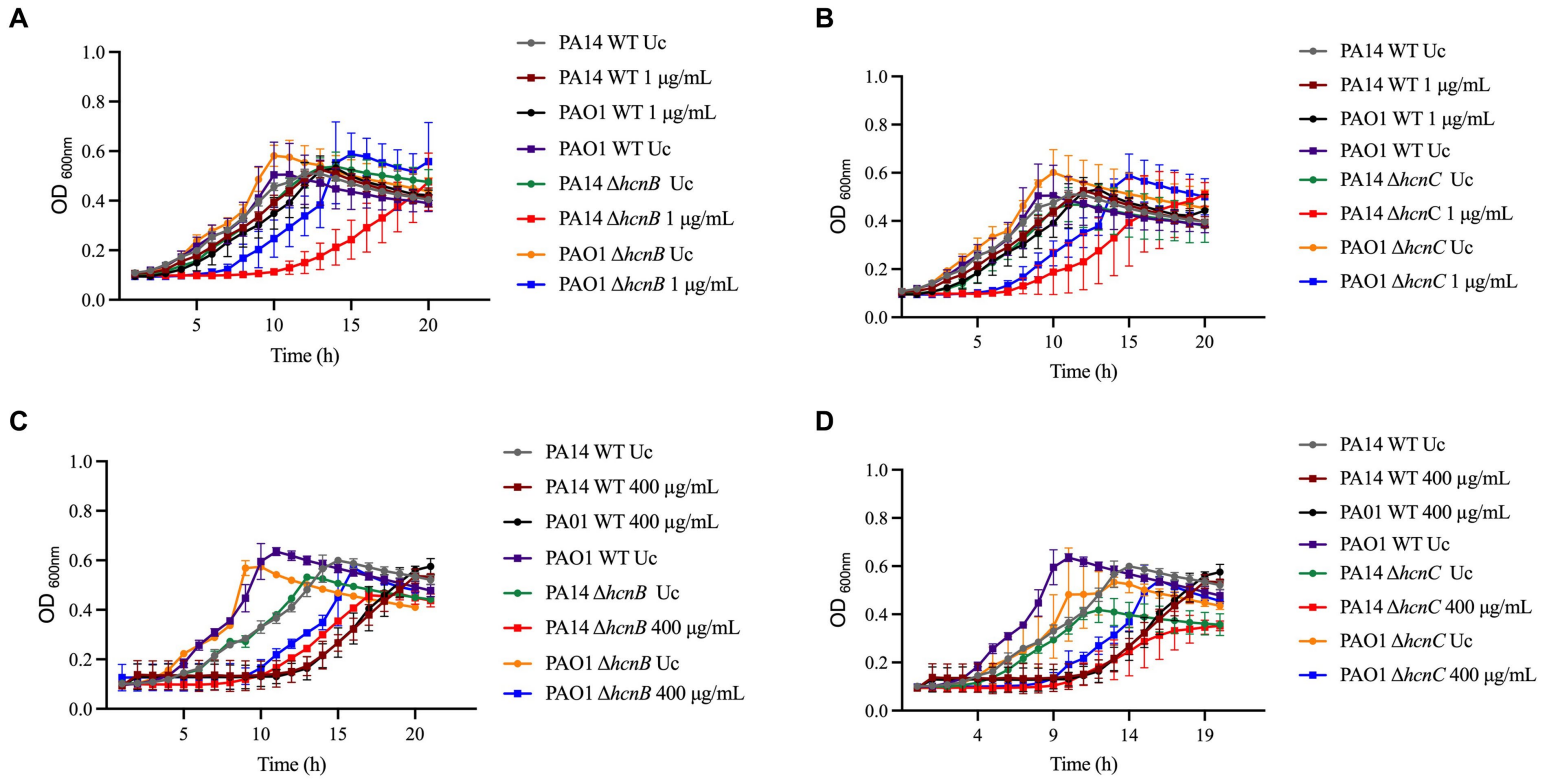
Susceptibility of *hcn* PA14 and PAO1 mutants to NaOCl and H_2_O_2_. Susceptibility to NaOCl of **(A)** Δ*hcnB* and **(B)** Δ*hcnC* PA14 and PAO1 mutants. Susceptibility to H_2_O_2_ of **(C)** Δ*hcnB* and **(D)** Δ*hcnC* PA14 and PAO1 mutants. Overnight cultures were grown in LB at 37°C and 220 rpm. Then, cells were washed twice, resuspended in BM2 minimal medium, and treated with NaOCl at 1 μg/mL or H_2_O_2_ at 400 μg/mL (final cell concentration of 1 × 10^8^ CFU/mL) for 20 h at 37°C. OD_600nm_ was recorded every hour using an epoch plate reader. WT: wild-type; Uc: untreated control.

Since HCN is produced by the *hcnABC* gene cluster in *P. aeruginosa* (Gilchrist et al., 2011), we evaluated if the absence of *hcnB* and *hcnC* also affects the susceptibility of *P. aeruginosa* to NaOCl by analyzing the growth of the corresponding PA14 and PAO1 Δ*hcnB* and Δ*hcnC* mutants at 1 μg/mL NaOCl for 20 h at 37°C. Like the Δ*hcnA* PA14 mutant, the corresponding PA14 and PAO1 Δ*hcnB* and Δ*hcnC* mutants showed an increase in susceptibility to NaOCl, presenting extended lag phase and reduced AUC compared to the WT strains at 1 μg/mL NaOCl. This phenotype seemed specific to NaOCl since these mutants did not present altered susceptibility to H_2_O_2_ at a final concentration of 400 μg/mL compared to WT strains (Figure 5; Table 4). These results confirm the importance of HCN production for *P. aeruginosa* survival under NaOCl stress conditions.

**Table 4:** Area under the curve (AUC) of NaOCl and H_2_O_2_ growth curves of *hcn Pseudomonas aeruginosa* mutants.

Gene name	*P. aeruginosa* strains	NaOCl		H_2_O_2_	
		Untreated	1 μg/mL	Untreated	400 μg/mL
	PAO1 WT	9.288 ± 0.875	7.946 ± 0.874	9.385 ± 0.747	5.542 ± 0.775
	PA14 WT	8.115 ± 0.983	7.310 ± 0.912	8.279 ± 0.748	4.253 ± 0.956
*hcnB*	PA2194 PW4739	7.507 ± 0.813	6.214 ± 1.074*	7.148 ± 0.092	5.199 ± 0.514
	PA14_36320	6.823 ± 0.504	3.626 ± 0.367*	6.739 ± 0.043	4.727 ± 0.161
*hcnC*	PA2195 PW4740	7.297 ± 112	5.727 ± 0.389*	6.718 ± 1.199	5.005 ± 0.395
	PA14_36310	5.389 ± 0.194	3.456 ± 0.294*	5.846 ± 0.337	3.407 ± 0.839

WT, wild-type. Data were analyzed by One-Way ANOVA and compared with the PA14 or PAO1 WT strains treated with 1 μg/mL NaOCl. *p < 0.05. AUC represents the average ± standard deviation of at least three independent experiments.

**Figure 6:**
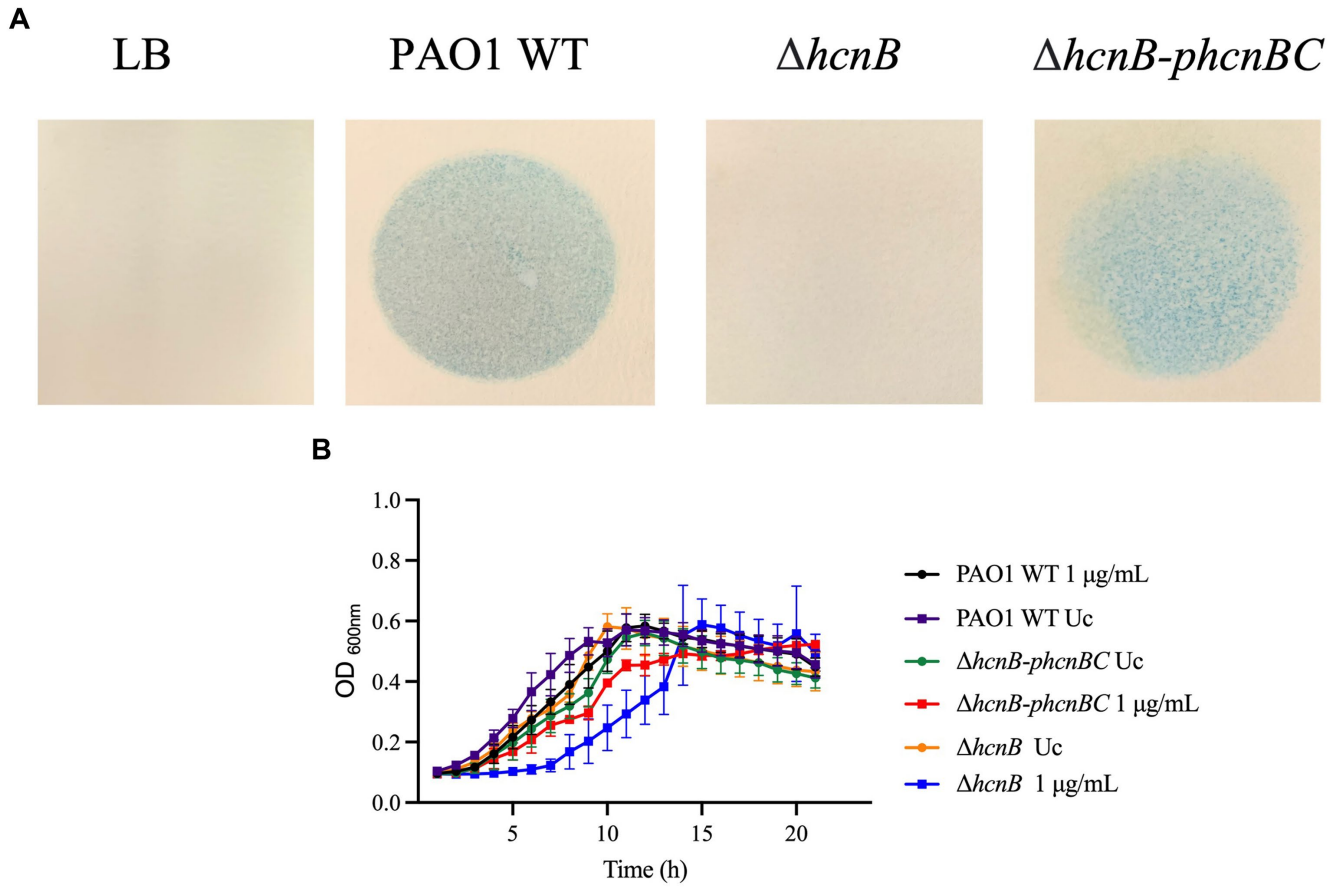
**(A)** Semiquantitative detection of HCN production by *P. aeruginosa* strains. cultures were grown in LB at 37°C and 220 rpm. Then, cells were washed twice and resuspended in LB. Two mL of overnight cultures were transferred to a small petri dish, which was covered by chromatography paper soaked in HCN detection reagent composed of copper (II) ethyl acetoacetate and 4,4′-methylenebis-(*N,N*-dimethylaniline) solubilized in chloroform. The small plates were placed in larger petri dish plates, which were covered. Production of volatile HCN was measured as the formation of blue color in the chromatography paper. The figures are representative of at least two independent experiments. **(B)** Susceptibility Δ*hcnB-phcnBC* complemented strain to NaOCl compared to PAO1 WT and PAO1 Δ*hcnB*. Overnight cultures were grown in LB at 37°C and 220 rpm. Then, cells were washed twice, resuspended in BM2 minimal medium, and treated with NaOCl at 1 μg/mL or (final cell concentration of 1 × 10^8^ CFU/mL) for 20 h at 37°C. OD_600nm_ was recorded every hour using an epoch plate reader. WT, wild-type; Uc, untreated control.

To test whether the increased susceptibility of the *hcn* mutants was due to the absence of HCN, we complemented the PAO1 mutant PA2194 PW4739 (Δ*hcnB*) by the transfer of the *phcnBC* plasmid, which expresses the genes *hcnBC*. Since both PA14 and PAO1 mutants presented increased susceptibility to NaOCl, we focused our analyzes on the PAO1 Δ*hcnB* mutant since this mutant has been characterized in a recent study (Létoffé et al., 2022). We assessed if the Δ*hcnB* mutant could be complemented by evaluating the release of HCN by our PAO1 strains using a semiquantitative method for HCN detection (Létoffé et al., 2022). When grown in LB for 24 h, both PAO1 WT and the Δ*hcnB-phcnBC* produced a detectable amount of HCN, while the Δ*hcnB* mutant did not produce HCN (Figure 6A).

Then, we conducted growth kinetic analyzes to determine whether the production of HCN by *P. aeruginosa* increases its resistance to NaOCl. As shown in Figure 6B, it took 6 h for the complemented strain Δ*hcnB-phcnBC* to reach the OD_600nm_ of 0.2, similar to the PAO1 WT strain. Furthermore, no statistical difference in growth was found for the AUC of Δ*hcnB-phcnBC* and PAO1 WT when treated with 1 μg/mL NaOCl (Table 5). Together, these results indicate that growth delay in response to NaOCl in the PAO1 Δ*hcnB* mutant could be complemented by the insertion of the *hcnBC-*producing plasmid *phcnBC*, demonstrating that HCN plays a role in the resistance of *P. aeruginosa* to NaOCl.

**Table 5:** Area under the curve (AUC) of NaOCl growth curves of PAO1-Δ*hcnB*- *phcnBC*.

	NaOCl	
	Untreated	1 μg/mL
PAO1 WT	9.288 ± 0.875	7.946 ± 0.874
Δ*hcnB*- *phcnBC*	7.21 ± 0.050	7.078 ± 0.038

AUC represents the average ± standard deviation of at least three independent experiments.

### 3.5. Characterization of HCN-mediated resistance to NaOCl in *Pseudomonas aeruginosa*

In order to provide further insight into the underlying mechanism of the HCN phenotype found in this study, we formulated two hypotheses: (i) HCN-related NaOCl resistance is mediated by cellular effects caused by HCN, or (ii) HCN acts as an extracellular metabolite, directly reacting with NaOCl and quenching its antimicrobial effect. In a previous study, Frangipani et al. (2014) investigated the effect of endogenously produced HCN on *P. aeruginosa* by transcriptomic analysis. The authors identified four *P. aeruginosa* genes that were repressed in response to endogenously produced HCN and 12 genes induced in response to HCN. To test if any of these HCN-controlled genes are involved in NaOCl resistance, we conducted growth analyzes by exposing *P. aeruginosa* PAO1 strains with mutations on the identified genes to 1 μg/mL NaOCl and measured their growth for 20 h. Among the mutants tested, only the mutant with a mutation in the PA4134 gene, which synthesizes a hypothetical protein with unknown function, presented statistically different AUC compared to the PAO1 WT strain treated with NaOCl (Supplementary Figure S2; Table 6). This mutant strain presented an AUC (5.785 ± 3.059) similar to Δ*hcnB* (6.214 ± 1.074). The PA4134 gene forms a gene cluster together with PA4133, with PA4133 being located upstream of PA4134;^1^ however, the PA4133 mutant did not show any significant difference in NaOCl susceptibility.

**Table 6:** Area under the curve (AUC) of NaOCl growth curves of *Pseudomonas aeruginosa* PAO1 and PA14 strains.

*P. aeruginosa* strains		Regulation by HCN^#^	AUC ± SD	
			Untreated	1 μg/mL NaOCl
PAO1 WT			9.288 ± 0.875	7.946 ± 0.874
PA14 WT			8.115 ± 0.983	7.310 ± 0.912
PA0433	PA0433 PW1792	↓	9.110 ± 0.503	7.682 ± 3.079
	PA14_05630		7.952 ± 0.268	7.624 ± 1.822
PA0434	PA0434 PW1793	↓	9.074 ± 0.311	6.904 ± 4.3
	PA14_05640		8.051 ± 0.323	6.828 ± 2.660
PA0435	PA0435 PW1795	↓	9.452 ± 0.502	7.005 ± 4.078
	PA14_05650		8.162 ± 0.38	7.520 ± 1.772
PA2299	PA2299 PW4885	↓	9.393 ± 0.366	8.354 ± 1.937
	PA14_15830		8.208 ± 0.190	7.609 ± 1.386
PA2328	PA2328 PW4927	↑	9.510 ± 0.164	8.565 ± 1.505
	PA14_41480		8.365 ± 0.455	7.592 ± 1.655
PA2329	PA14_02330	↑	8.137 ± 0.621	7.728 ± 1.317
PA2330	PA2330 PW4930	↑	9.368 ± 0.305	8.775 ± 0.830
	PA14_34490		7.709 ± 1.227	7.802 ± 1.517
PA2331	PA2331 PW7998	↑	9.490 ± 0.232	7.544 ± 1.839
	PA14_10540		8.232 ± 0.359	7.617 ± 0.615
PA3022	PA3022 PW6063	↑	9.181 ± 0.331	7.228 ± 2.425
	PA14_24980		8.038 ± 0.505	6.325 ± 1.978
PA4129	PA4129 PW7993	↑	9.113 ± 0.397	6.482 ± 1.977
PA4130	PA4130 PW7996	↑	9.290 ± 0.083	7.835 ± 0.936
	PA14_10550		6.401 ± 0.398	6.176 ± 0.577
PA4131	PA4131 PW7998	↑	9.367 ± 0.115	7.331 ± 0.316
	PA14_10540		8.238 ± 0.105	7.278 ± 0.483
PA4132	PA4132 PW8001	↑	8.879 ± 0.457	7.095 ± 1.207
	PA14_10530		7.652 ± 0.176	6.651 ± 0.933
PA4133	PA4133 PW8002	↑	9.595 ± 0.243	7.832 ± 0.309
	PA14_10500		6.479 ± 0.112	5.895 ± 0.227
PA4134	PA4134 PW8004	↑	9.748 ± 0.522	5.785 ± 3.059*

^#^Gene regulation by endogenously produced HCN by transcriptomic analysis conducted by Frangipani et al. (2014). WT, wild-type. Data were analyzed by One-Way ANOVA and compared with the PA14 or PAO1 WT strains treated with 1 μg/mL NaOCl. ↓ gene downregulated and ↑ upregulated in the study conducted by Frangipani et al. (2014). *p < 0.05. AUC represents the average ± standard deviation of at least three independent experiments.

**Figure 7:**
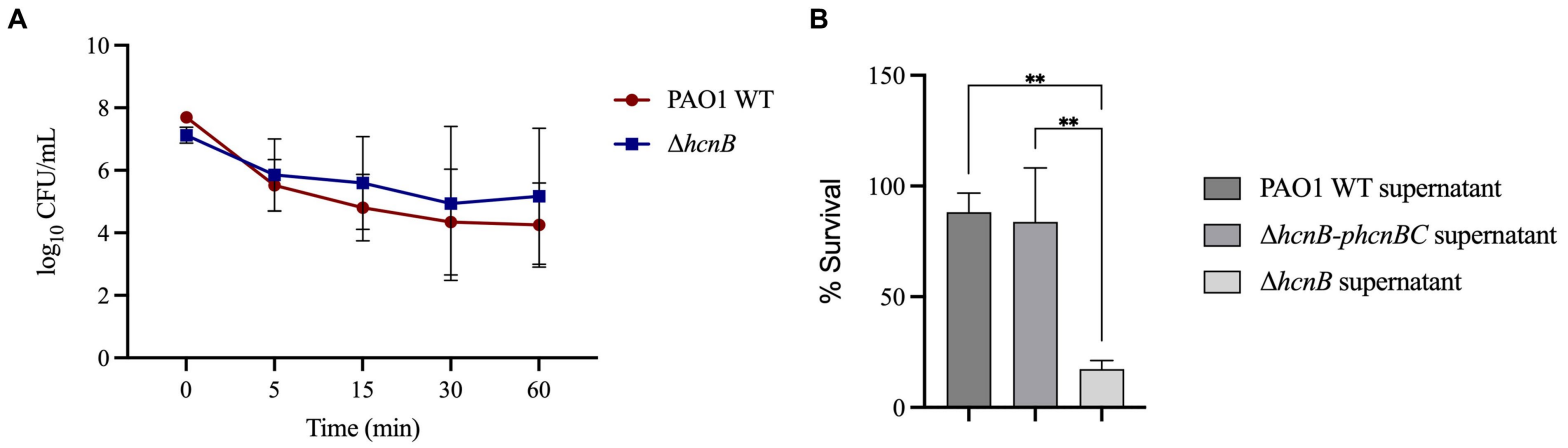
Effect of exogenous HCN on the susceptibility of *P. aeruginosa* to NaOCl. **(A)** PAO1 WT and Δ*hcnB* were grown overnight, washed twice, and resuspended in PBS. The OD_600nm_ was adjusted to 0.1, and the cell suspension was treated with 2 μg/mL NaOCl for 5, 15, 30, and 60 min. Then, CFU counts were determined by the drop plate method. **(B)** Δ*hcnB*, PAO1 WT, and Δ*hcnB-phcnBC* were grown overnight in BM2, and the supernatant was collected by centrifugation followed by filtration in a 0.22 μm filter. Δ*hcnB* overnight cells were resuspended in each supernatant to an OD_600nm_ of 0.1, treated with NaOCl at 4 μg/mL, and the CFU counts were determined by the drop plate method. ** *p* < 0.01.

We then tested if the metabolite HCN itself would directly react with NaOCl and quench its toxic effect. For this, we first analyzed if removing HCN from the medium would change the susceptibility of Δ*hcnB* and PAO1 WT strains by evaluating the kill kinetics in response to NaOCl. Cells grown overnight were washed twice, resuspended in PBS to remove the HCN from the medium, and NaOCl was added to a final concentration of 2 μg/mL. The absence of HCN in the medium did not provoke a difference in the NaOCl susceptibility of PAO1 WT and Δ*hcnB* (Figure 7A), in which no statistical difference was found for the cell concentration over time for both strains. Overall, the growth analyzes and kill-kinetic results suggested that the susceptibility phenotype found for Δ*hcnB* is likely not due to a cellular effect.

Next, we aimed to complement the susceptibility phenotype of Δ*hcnB* with the supernatant from PAO1 WT and the complemented strain Δ*hcnB*-*phcnBC.* We used the culture supernatant of HCN-producing strains instead of the pure chemical HCN due to the high toxicity of HCN and its related forms [e.g., sodium cyanide (NaCN) and potassium cyanide (KCN)]. For this, we resuspended overnight Δ*hcnB* cells in the supernatants of Δ*hcnB*, PAO1 WT, and Δ*hcnB*-*phcnBC* grown in BM2 minimal medium. Then, the bacterial suspensions were treated with NaOCl at 4 μg/mL for 60 min. Figure 7B shows that the addition of PAO1 WT and Δ*hcnB*-*phcnBC* supernatants to cells of Δ*hcnB* complemented the Δ*hcnB* phenotype, and Δ*hcnB* cells showed increased resistance to NaOCl (percentage survival of 88 and 83%, respectively) compared to the Δ*hcnB* cells resuspended in Δ*hcnB* supernatant (17% survival). These results suggest that HCN reacts with NaOCl, quenching its lethal effect.

## 4. Discussion

Bacteria have developed several mechanisms to mitigate the harmful and often irreversible damage caused by oxidizing agents. Most of these resistance mechanisms are not specific but rather provide a general defense against a broad range of oxidizing agents. In this context, the knowledge of HOCl-specific responses is still limited (Gray et al., 2013; da Cruz Nizer et al., 2020). Among the HOCl responses described so far, ATP-independent chaperones are considered the immediate response against HOCl since they are readily activated by the oxidation of amino acid residues. These enzymes are essential for HOCl response since they do not require the expression of sensor mechanisms, which takes a long time compared to the fast action of HOCl on proteins (Goemans and Collet, 2019; Sultana et al., 2020). Other mechanisms include the activation of the transcriptional regulators HypT, NemR, and RclR and the formation of biofilms (Gray et al., 2013; Strempel et al., 2017; Da Cruz Nizer et al., 2020).

In this study, we demonstrated that the loss of KatA, KatE, AhpC, AhpF, and MsrA increases the susceptibility of *P. aeruginosa* PAO1 and PA14 to NaOCl compared to the WT strains, showing that these H_2_O_2_ responses are employed by this bacterium as a general response against oxidizing agents. We then identified 48 mutants with increased susceptibility to NaOCl and characterized 10 mutants in more detail. Among them, we found that HCN acts as a scavenger molecule and increases the survival of *P. aeruginosa* in the presence of NaOCl.

Most oxidative stress responses have been characterized for H_2_O_2_ and other ROS, while their roles in RCS and NaOCl resistance remain mostly unknown. Therefore, in the first part of this study, we showed by growth kinetics analyzes that well-known H_2_O_2_ responses are also involved in the survival of *P. aeruginosa* to NaOCl. Catalases and peroxidases are specialized enzymes that convert H_2_O_2_ into less toxic species (i.e., H_2_O + O_2_ and alcohol + H_2_O, respectively; Cavinato et al., 2020). These enzymes are widely distributed among bacteria (Yuan et al., 2021) and are the primary response against oxidative stress (Romsang et al., 2013). *P. aeruginosa* has three catalases (*katA, katB,* and *katE*) and four alkyl hydroperoxide reductases (*ahpA*, *ahpB*, *ahpCF*, and *ohr*), in which KatA is considered the main catalase and its expression is controlled by various systems, such as OxyR, quorum sensing, and ANR (anaerobic regulator; Heo et al., 2010; Su et al., 2014). KatA and AhpA are continually expressed during bacterial growth, implying their importance as a defense not only during harmful conditions but also against endogenously produced ROS (Ochsner et al., 2000; Lee et al., 2005). Many studies have shown that the production of these detoxifying systems is upregulated by oxidizing agents in *P. aeruginosa*, such as H_2_O_2_ (Salunkhe et al., 2002; Palma et al., 2004; Chang et al., 2005; Small et al., 2007a,b), NaOCl (Small et al., 2007a,b; Groitl et al., 2017), hypobromous acid (Groitl et al., 2017), hypothiocyaous acid (Groitl et al., 2017) and peracetic acid (Chang et al., 2005). Furthermore, *P. aeruginosa katA*, *katB*, *ahpB,* and *oxyR* mutant strains were consistently more susceptible to H_2_O_2_ than the WT strains (Ochsner et al., 2000; Lee et al., 2005), corroborating our results. This susceptibility phenotype of *kat* and *ahp* mutants was also reported for other bacterial strains, such as *Vibrio cholerae* (Wang et al., 2012) and *Stenotrophomonas maltophilia* (Li et al., 2020). In the context of HOCl resistance, little has been explored on the roles of these enzymes in the resistance of *P. aeruginosa* toward this oxidant. The importance of detoxifying enzymes such as catalases has been described for *Escherichia coli* and *Helicobacter pylori* (Dukan and Touati, 1996; Benoit and Maier, 2016), in which catalases are considered a ubiquitous enzymes with quenching ability toward oxidizing agents in general (Benoit and Maier, 2016), corroborating our findings for *P. aeruginosa*. Considering that proteins are the main target of HOCl, another important stress response mechanism is the protein repair system Msr. *P. aeruginosa* and most bacterial species have two highly conserved Msr systems: MsrA and MsrB (Romsang et al., 2013). As for KatA and AhpA, MsrA is expressed during all growth phases, while MsrB is overproduced under oxidative stress conditions (Romsang et al., 2013). In accordance with our PA14 susceptibility results, PAO1 *msrA* and *msrB* mutants presented increased susceptibility to H_2_O_2_ and NaOCl in the study by Romsang et al. (2013). Hence, our findings report the involvement of *kat*, *ahp,* and *msr* genes in NaOCl resistance, adding to the previously described function of these response mechanisms.

We then performed a genome-wide mutant library screening of the PA14 transposon mutant library (Liberati et al., 2006) to find mutants with increased susceptibility to NaOCl. We were able to identify 48 genes with reduced MIC values toward NaOCl compared to PA14 WT, and we characterized 10 mutants (disrupted *nrdJa*, *bvlR*, *hcnA*, *orn*, *sucC*, *cysZ*, *nuoJ*, PA4166, *opmQ,* and *thiC* gene, respectively) in more detail (Table 2). Library screenings allow access to a large number of mutants carrying specific genetic alterations; therefore, researchers can simultaneously screen multiple mutants for the phenotype of interest in a short period. This approach is valuable for uncovering novel genes and pathways that contribute to bacterial resistance (Moser et al., 2014). Among them, two mutant strains, Δ*cysZ* and Δ*opmQ*, lacked genes involved in the transport of small molecules. The first one (Δ*cysZ*) has a mutation in a sulfate uptake protein. Proteins, mainly the sulfur-containing ones, are the main target of HOCl in the cells (Da Cruz Nizer et al., 2020); therefore, due to the reduction of the amount of sulfur in the cells due to its reaction with HOCl, the transport of this compound to the cells seems to be necessary (Farrant et al., 2020). The upregulation of transport and metabolism of sulfur genes by HOCl has also been described for *E. coli* and *Salmonella enterica* Serovars Enteritidis (Wang et al., 2009, 2010). Overexpression of genes involved in the transport of small molecules (Small et al., 2007a,b) has been detected by transcriptomic studies and has been implicated in the need of cells to allow the entry or exit of metabolites, such as toxic HOCl-by-products and compounds needed for cell metabolism (Albrich et al., 1986).

In accordance with previous reports about the oxidation of DNA by HOCl (Prütz, 1996), we found the *nrdJa* gene, which encodes for a ribonucleotide reductase and was previously reported to be involved in DNA repair (Torrents et al., 2005). NrdJa is crucial for growth under anaerobic conditions (Wu et al., 2005; Filiatrault et al., 2013) and was upregulated after ciprofloxacin exposure (Cirz et al., 2006). Furthermore, Crespo et al. (2017) also found increased transcription of *nrdJ* under H_2_O_2_ stress, supporting our findings (Crespo et al., 2017). Ribonucleotide reductases have also been shown to be used by many other bacteria, such as *Bifidobacterium longum* (Zuo et al., 2018), *Bacillus subtilis* (Castro-Cerritos et al., 2017) and *E. coli* (Monje-Casas et al., 2001) as a response mechanism to oxidative stress, mainly H_2_O_2_.

Considering the high metabolic diversity of *P. aeruginosa*, many genes and pathways remain to be explored regarding their secondary effects and possible roles in resistance. In this context, we identified two genes involved in energy metabolism (i.e., *sucC* and *nuoJ*) and one implicated in thiamine biosynthesis (*thiC*). Thiamine, for example, has been explored as a target in the development of antibiotics (Lünse et al., 2014; Kim et al., 2020), while energy metabolism genes, such as *sucC* and *nuoH*, *nuoM,* and *nuoN* have been shown to be downregulated in response to tobramycin in *A. baumannii* (Kashyap et al., 2022). Furthermore, transcriptomic studies have shown the downregulation of energy production genes in cells under HOCl (Small et al., 2007a,b). However, their contribution to oxidative stress remains to be elucidated.

In our screening, we also identified genes involved in the pathogenicity of *P. aeruginosa* by controlling virulence factors and resistance production (*bvlR* and *orn*). BvlR is a transcriptional repressor that belongs to the LysR-type transcriptional regulator (LTTR) family. It is upregulated during exposure to epithelial cells (Frisk et al., 2004) and controls several virulence factors in *P. aeruginosa*, indicating a role in the pathogenicity of this bacterium. BvlR represses the expression of the type 3 secretion system (T3SS), *cupA*-associated fimbrial-based surface attachment, and toxin A. Furthermore, it was shown to promote tight microcolony formation, which is associated with the formation of biofilms in the lung of cystic fibrosis (*CF*) patients (McCarthy et al., 2014). The oligorribonuclease Orn also affects T3SS production (Chen et al., 2016) and contributes to bacterial resistance to fluoroquinolones by a pyocin-mediated mechanism (Chen et al., 2017) and aminoglycosides, β-lactams and oxidative stress by influencing the translation of *katA* mRNA (Xia et al., 2019).

Another finding in our screening was a mutant with a disrupted *hcnA* gene. The absence of HCN in our *P. aeruginosa* mutant strains increased their susceptibility to NaOCl, while the complementation of Δ*hcnB* with an HCN-producing plasmid recovered the phenotype in our growth kinetic analyzes. HCN is a toxic volatile secondary metabolite as it has no apparent function in primary metabolism and is produced at later stages during the exponential phase and offers an advantage for the producing strain, which is tolerant to it (Blumer and Haas, 2000). It is synthesized by many bacterial genera, including *Alcaligenes*, *Aeromonas*, *Bacillus*, *Pseudomonas*, and *Rhizobium* (Blumer and Haas, 2000; Abd El-Rahman et al., 2019). In *P. aeruginosa*, HCN is synthesized by the HCN synthase, encoded by the *hcnABC* operon, and regulated by quorum sensing and the ANR regulator (Pessi and Haas, 2000). *P. aeruginosa* cultures produce up to 300 μM of HCN by the decarboxylation of glycine, and the *hcnABC* operon is induced by low oxygen (Blumer and Haas, 2000) and high cell density, with maximum production at the end of the exponential phase (Blier et al., 2012). The toxic effect of HCN is due to the inhibition of cytochrome c oxidase, impairing cell oxygen consumption and energy production (Zuhra and Szabo, 2022). *P. aeruginosa* has two systems to avoid HCN intoxication. One involves a cyanide-insensitive terminal oxidase, CioAB, which allows aerobic respiration in the presence of cyanide (Cunningham et al., 1997) and the other system is the enzyme rhodonase, which forms thiocyanate by the reaction with HCN (Cipollone et al., 2007).

HCN has been detected in the breath (Enderby et al., 2009; Smith et al., 2013) and the sputum and lung of *CF* patients (Ryall et al., 2008; Sanderson et al., 2008), suggesting that *P. aeruginosa* could also employ this metabolite as a virulence factor to increase its pathogenicity. Due to its high toxicity, HCN also exerts a toxic effect on non-producing strains (Zdor, 2015; Biswas and Götz, 2022). In this context, HCN produced by *P. aeruginosa* has been shown to control the growth of *S. aureus*, contributing to *P. aeruginosa* competition (Létoffé et al., 2022). Here we show that HCN also contributes to the survival of *P. aeruginosa* to the strong antioxidant NaOCl. Recently, studies have shown the effect of signaling molecules on the resistance profile of bacterial species (Li et al., 2022). Therefore, to investigate the HCN-mediated response to NaOCl in *P. aeruginosa,* we first tested the hypothesis that the production of HCN induces cellular mechanisms that, in turn, activate resistance mechanisms. For instance, indole produced by bacterial species induces bacterial resistance endogenously and exogenously by many mechanisms, including efflux pump regulation, biofilm formation, and induction of the persister state (Li et al., 2022). We then tested a list of *P. aeruginosa* genes that were up or down-regulated under endogenously produced HCN (Frangipani et al., 2014). Except for the gene PA4134, no NaOCl susceptibility was found for the mutant strains tested, suggesting that the NaOCl phenotype found in this study is likely not due to cellular regulation.

We then assessed if the NaOCl susceptibility found was due to the reaction of HCN with NaOCl and found evidence supporting the scavenger effect of HCN in the presence of NaOCl, quenching the toxic effect of this oxidizing agent. Due to its high reactivity, HOCl is known to react rapidly with sulfur- and nitrogen-containing compounds, producing chlorinated derivatives with impaired functions (Winterbourn, 1985; Pattison et al., 2012). In this context, the reaction between HCN and HOCl, the active ingredient of NaOCl in aqueous solution (Fukuzaki, 2006), forms CO_2_, N_2_, and HCl. However, due to its toxicity, we could not evaluate if adding HCN to *P. aeruginosa* would rescue the NaOCl-susceptibility phenotype found for the Δ*hcnB* mutant, and we used different supernatants from WT and HCN-deficient strains as an alternative approach.

It is believed that the production of HCN by bacteria serves various purposes, including defense mechanisms, helping the bacteria compete with other microorganisms for resources, and antimicrobial effect, inhibiting the growth of other microorganisms. Here, we have identified a new role for HCN produced by pathogenic bacteria as a NaOCl scavenger molecule, contributing to bacterial resistance under NaOCl stress conditions. We hypothesize that HCN is produced in the context of *P. aeruginosa* infection, such as wound infection in which the environment presents low oxygen levels, favoring anaerobic microorganisms (Versey et al., 2021), and helping in the fight against oxidative stress produced by the immune system or from exogenous sources. Many bacteria produce a wide array of virulence factors and secreted metabolites. Many of these secreted molecules have been shown to quench and neutralize the toxic effect of oxidizing agents. For example, melanin produced by bacteria is a free radical scavenger (Sichel et al., 1991; Agodi et al., 1996) that protects bacterial cells against oxidative stress (Rodríguez-Rojas et al., 2009; Thippakorn et al., 2018). The search for molecules that act as scavengers for antimicrobial agents or immune system factors and understanding these metabolites help develop new strategies to eradicate *P. aeruginosa* and fight infections and the spread of this bacterium.

Of note, due to the high reactivity of NaOCl and its active ingredient, HOCl, the chemistry behind the formation and consumption of RCS in media is complex (Peskin et al., 2005; Pattison and Davies, 2006). For instance, rich media such as LB have been shown to completely quench the oxidizing effect of HOCl, while in some buffers such as PBS, no change in the levels of RCS was detected (Ashby et al., 2020). In this context, in minimal growth media containing amine, chloramines can be formed by the chlorination of amino groups by the chlorine present in HOCl (Pál Fehér et al., 2019). Therefore, the amount of HOCl and other RCS will depend on the type of media used and their ability to quench HOCl as well as to produce other RCS.

## 5. Conclusion

Although much effort has been made to uncover the mechanisms employed by bacteria to resist oxidative stress, most of the studies have focused on H_2_O_2_, and the knowledge on adaption to RCS, including NaOCl, is still in its infancy. Our PA14 mutant library screening identified 48 genes and showed that *P. aeruginosa* relies on diverse mechanisms to survive the potent and often irreversible stress caused by NaOCl. Among them, we identified the *hcnA* gene and showed that HCN contributes to the resistance of *P. aeruginosa* by quenching the toxic effect of NaOCl. To our knowledge, this is the first study reporting the roles of HCN in NaOCl resistance.

## Data availability statement

The original contributions presented in the study are included in the article/Supplementary material, further inquiries can be directed to the corresponding author.

## Author contributions

WSdCN: Conceptualization, Formal analysis, Investigation, Methodology, Project administration, Visualization, Writing – original draft, Writing – review & editing. MEA: Formal analysis, Investigation, Methodology, Writing – original draft. VI: Formal analysis, Investigation, Methodology, Writing – original draft. CB: Methodology, Writing – original draft, Investigation. JO: Methodology, Writing – original draft, Conceptualization, Funding acquisition, Project administration, Resources, Supervision, Writing – review & editing.

## Footnotes

^1^pseudomonas.com
