# Supplementary material for: The secondary metabolite hydrogen cyanide protects Pseudomonas aeruginosa against sodium hypochlorite-induced oxidative stress
Source: Front Microbiol. 2023 Nov 16;14:1294518. doi: 10.3389/fmicb.2023.1294518 (PMC10687435; doi:10.3389/fmicb.2023.1294518)
Supplement: Supplementary file 1 [file Data_Sheet_1.docx]

Supplementary Material

# Supplementary Figures and Tables

## Supplementary Figures

| **(A)** | **(B)** |
| --- | --- |
| 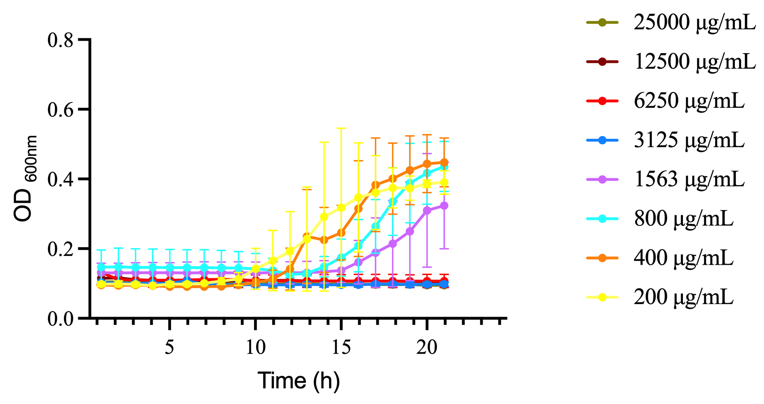 | 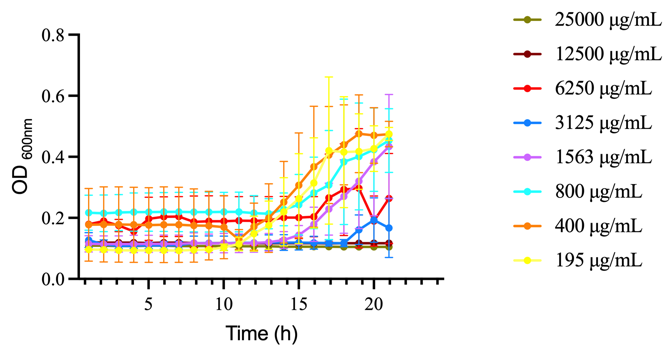 |

**Supplementary Figure S1.** Susceptibility of **(A)** PA14 and **(B)** PAO1 wild-type strains to different concentrations of H_2_O_2_ (i.e., 200, 400, 800, 1563, 3125, 6250, 12500, 25000 μg/mL). Overnight cultures were grown in LB at 37^o^C and 220 rpm. Then, cells were washed twice, resuspended in BM2 minimal media, and treated with H_2_O_2_ (final cell concentration of 1 × 10^8^ CFU/mL) for 20 h at 37^o^C. OD_600nm_ was recorded every hour using an epoch plate reader.

| **A** | **B** |
| --- | --- |
| **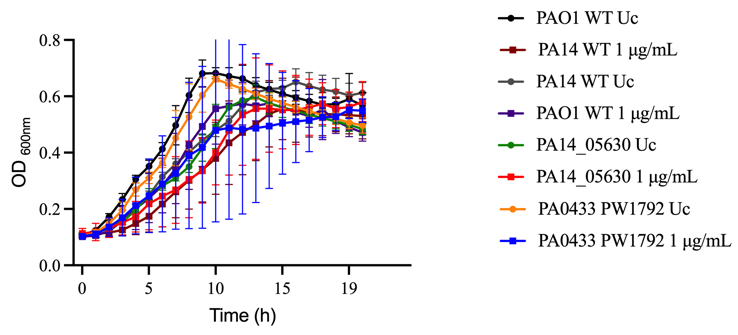** | **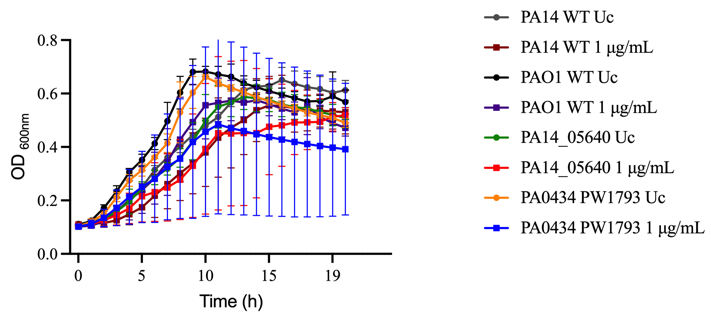** |
| **C** | **D** |
| **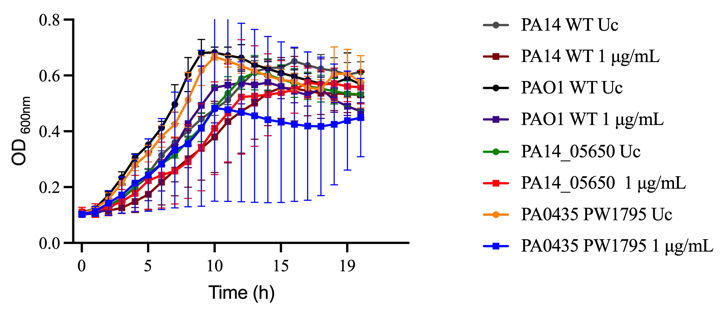** | **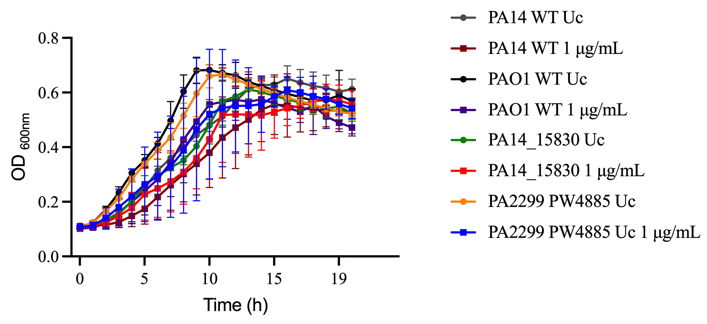** |
| **E** | **F** |
| **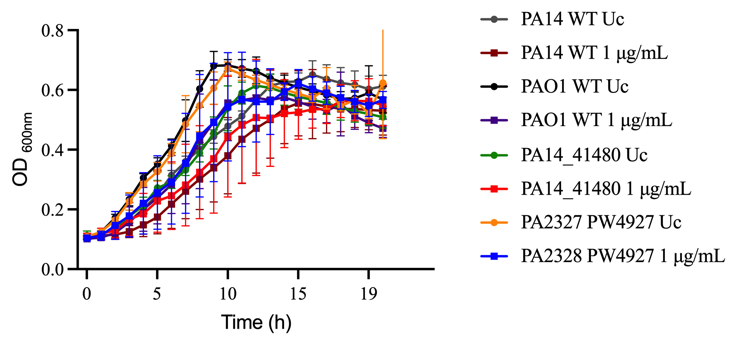** | **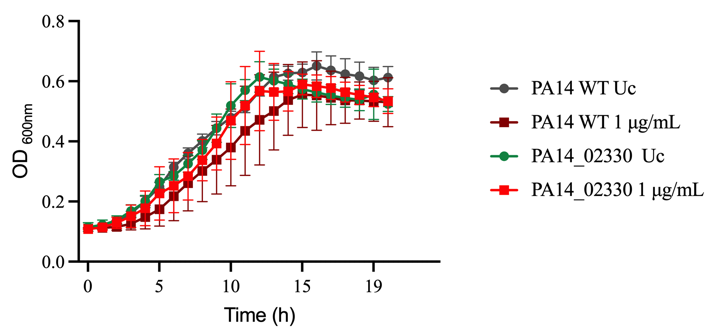** |
| **G** | **H** |
| **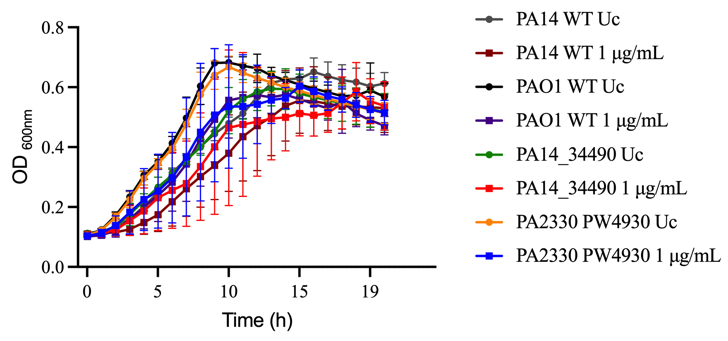** | **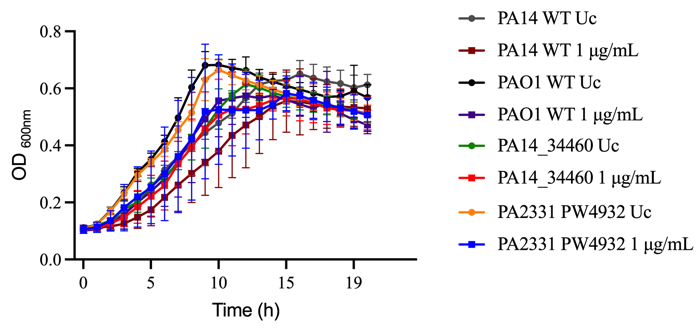** |
| **I** | **J** |
| **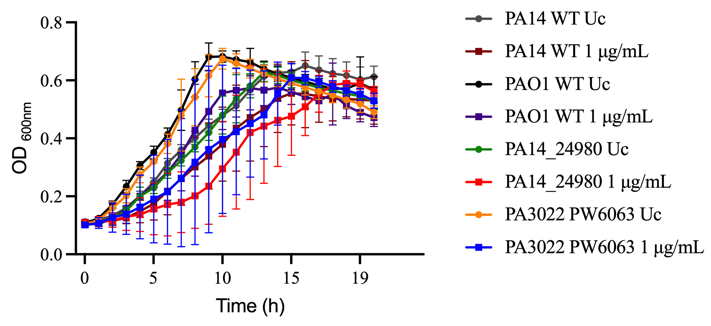** | **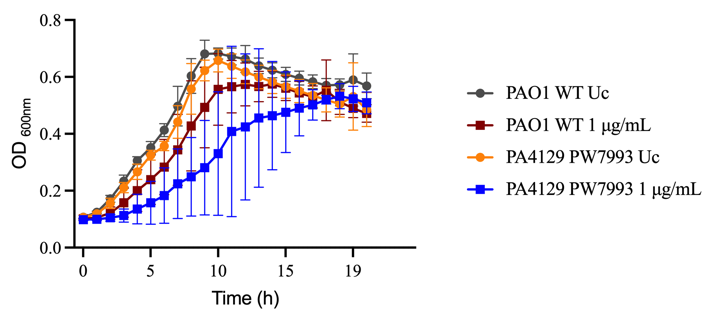** |
| **K** | **L** |
| **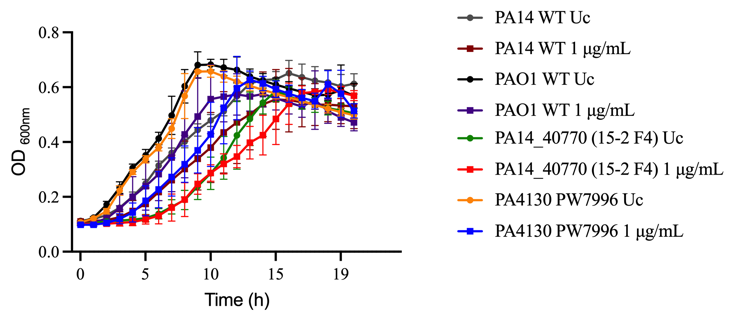** | **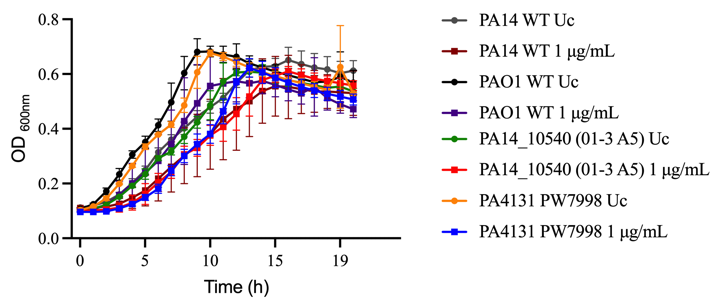** |
| **M** | **N** |
| **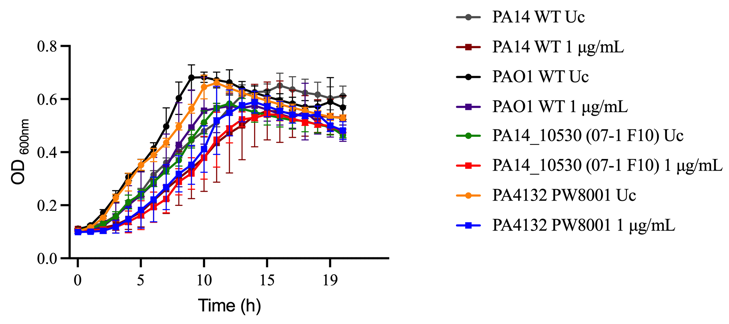** | **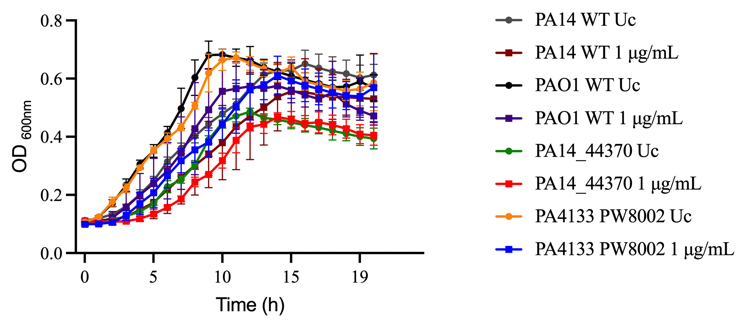** |
| **O** |  |
| **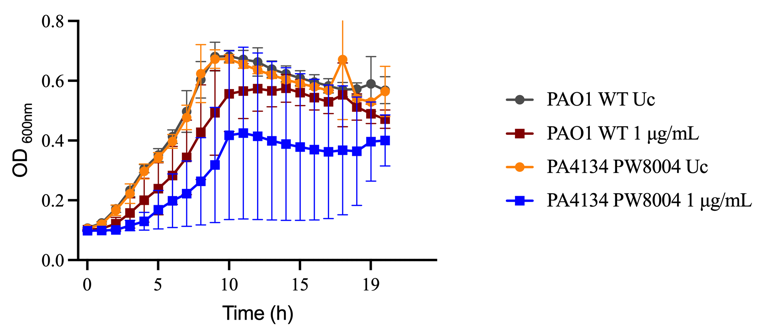** |  |

**Supplementary Figure S2.** Susceptibility of PA14 and PAO1 genes that were up or downregulated by endogenous hydrogen cyanide (HCN) to NaOCl. Overnight cultures were grown in LB at 37^o^C and 220 rpm. Then, cells were washed twice, resuspended in BM2 minimal media, and treated with NaOCl at 1 μg/mL (final cell concentration of 1 × 10^8^ CFU/mL) for 20 h at 37^o^C. OD_600nm_ was recorded every hour using an epoch plate reader. **(A)** PA0433; **(B)** PA0434; **(C)** PA0435; **(D)** PA2299; **(E)** PA2328; **(F)** PA2329; **(G)** PA2330; **(H)** PA2331; **(I)** PA3022; **(J)** PA4129; **(K)** PA4130; **(L)** PA4131; **(M)** PA4132; **(N)** PA4133; **(O)** PA4134. WT: wild-type; Uc: untreated control.

## Supplementary Tables

**Supplementary Table S1.** Strains and plasmids used in this study.

| **Strain or Plasmid** | | **Relevant characteristics** | **Reference or Source** |
| --- | --- | --- | --- |
| **PA number and ID** | **Gene name** |  |  |
| ***P. aeruginosa* PA14 strains** | |  |  |
| PA14 WT |  | Wild-type strain | (Rahme et al., 1995) |
| PA14_09150 29943 | Δ*katA* |  | (Liberati et al., 2006) |
| PA14_36810 53653 | Δ*katE* |  |  |
| PA14_01710 55227 | Δ*ahpC* |  |  |
| PA14_01720 37932 | Δ*ahpF* |  |  |
| PA14_66330 42189 | Δ*msrA* |  |  |
| PA14_27230 56518 | Δ*ohrR* |  |  |
| PA14_72540 27304 | Δ*nrdJa* |  |  |
| PA14_26880 27401 | Δ*bvlR* |  |  |
| PA14_36330 42284 | Δ*hcnA* |  |  |
| PA14_65410 45346 | Δ*orn* |  |  |
| PA14_43950 46221 | Δ*sucC* |  |  |
| PA14_53330 46544 | Δ*cysZ* |  |  |
| PA14_29900 47870 | Δ*nuoJ* |  |  |
| PA14_09990 56522 |  | Hypothetical protein |  |
| PA14_33750 56649 | Δ*opmQ* |  |  |
| PA14_65740 55316 | Δ*thiC* |  |  |
| PA14_36320 40718 | Δ*hcnB* |  |  |
| PA14_36310 28000 | Δ*hcnC* |  |  |
| PA14_05630 46761 |  | Hypothetical protein |  |
| PA14_05640 39916 |  | Hypothetical protein |  |
| PA14_05650 32396 |  | Hypothetical protein |  |
| PA14_15830 33205 | Δ*gntR* |  |  |
| PA14_41480 23227 |  | Hypothetical protein |  |
| PA14_02330 25450 | Δ*atsC* |  |  |
| PA14_34490 26462 |  | Acyl-CoA dehydrogenase |  |
| PA14_10540 26848 |  | Iron-sulfur cluster-binding protein |  |
| PA14_24980 28435 |  | Hypothetical protein |  |
| PA14_10550 37691 | Δ*nirA* |  |  |
| PA14_10540 6852 |  | Iron-sulfur protein |  |
| PA14_10530 35804 | Δ*mpaR* |  |  |
| PA14_10500 40170 | Δ*ccoN* |  |  |
|  |  |  |  |
| ***P. aeruginosa* PAO1 strains** | |  |  |
| PAO1 WT |  | Wild-type strain | (Stover et al., 2000) |
| PA4236 PW8190 | Δ*katA* |  | (Jacobs et al., 2003) |
| PA2147 PW4679 | Δ*katE* |  |  |
| PA0139 PW1231 | Δ*ahpC* |  |  |
| PA0140 PA1233 | Δ*ahpF* |  |  |
| PA5018 PW9426 | Δ*msrA* |  |  |
| PA2849 PW5780 | Δ*ohrR* |  |  |
| PA5497 PW10298 | Δ*nrdJa* |  |  |
| PA2877 PW5830 | Δ*bvlR* |  |  |
| PA4951 PW9335 | Δ*orn* |  |  |
| PA0846 PW2541 | Δ*cysZ* |  |  |
| PA2645 PW5426 | Δ*nuoJ* |  |  |
| PA4166 PW8062 |  |  |  |
| PA2391 PW5021 | Δ*opmQ* |  |  |
| PA4973 PW9367 | Δ*thiC* |  |  |
| PA2194 PW4739 | Δ*hcnB* |  |  |
| PA2195 PW4740 | Δ*hcnC* |  |  |
| PA0433 PW1792 |  | Hypothetical protein |  |
| PA0434 PW1793 |  | Hypothetical protein |  |
| PA0435 PW1795 |  | Hypothetical protein |  |
| PA2299 PW4885 | Δ*gntR* |  |  |
| PA2328 PW4927 |  | Hypothetical protein |  |
| PA2330 PW4930 |  | Acyl-CoA dehydrogenase |  |
| PA2331 PW7998 |  | Iron-sulfur cluster-binding protein |  |
| PA3022 PW6063 |  | Hypothetical protein |  |
| PA4129 PW7993 |  | Hypothetical protein |  |
| PA4130 PW7996 | Δ*nirA* |  |  |
| PA4131 PW7998 |  | Iron-sulfur protein |  |
| PA4132 PW8001 | Δ*mpaR* |  |  |
| PA4133 PW8002 | Δ*ccoN* |  |  |
| PA4134 PW8004 |  | Hypothetical protein |  |
| Δ*hcnB-phcnBC* |  | PAO1 Δ*hcnB* mutant complemented with *phcnBC* plasmid; Kan resistance; inducible by 2mM sodium benzoate | This study |
| **Plasmid** |  |  |  |
| *phcnBC* |  | pSEVA238 plasmid expressing *hcnBC* | (Létoffé et al., 2022) |

**Supplementary Table S2.** NaOCl-sensitive PA14 mutants (MIC of 0.5 and 1 µg/mL) identified in the preliminary screening and MIC testing.

| PA14 Locus name | PAO1 homolog | Gene name | Gene description | Functional category | MIC (µg/mL)^*^ |
| --- | --- | --- | --- | --- | --- |
| PA14_72540 | PA5497 | *nrdJa* | Putative ribonucleotide reductase | Nucleotide biosynthesis and metabolism | 1 |
| [PA14_36520](http://pa14.mgh.harvard.edu/cgi-bin/pa14/view_gene.cgi?GeneID=GID4528) | [PA2171](http://pseudomonas.com/AnnotationByPAu.asp?PA=PA2171) |  | Conserved hypothetical protein | Hypothetical protein | 1 |
| [PA14_11030](http://pa14.mgh.harvard.edu/cgi-bin/pa14/view_gene.cgi?GeneID=GID912) | [PA4088](http://pseudomonas.com/AnnotationByPAu.asp?PA=PA4088) |  | Putative aminotransferase | Transport of small molecules | 1 |
| [PA14_51300](http://pa14.mgh.harvard.edu/cgi-bin/pa14/view_gene.cgi?GeneID=GID1875) | [PA1007](http://pseudomonas.com/AnnotationByPAu.asp?PA=PA1007) |  | Conserved hypothetical protein | Hypothetical protein | 1 |
| [PA14_18140](http://pa14.mgh.harvard.edu/cgi-bin/pa14/view_gene.cgi?GeneID=GID2627) | [PA3569](http://pseudomonas.com/AnnotationByPAu.asp?PA=PA3569) | *mmsB* | 3-hydroxyisobutyrate dehydrogenase | Carbon compound catabolism | 1 |
| [PA14_03150](http://pa14.mgh.harvard.edu/cgi-bin/pa14/view_gene.cgi?GeneID=GID3561) | [PA0255](http://pseudomonas.com/AnnotationByPAu.asp?PA=PA0255) |  | Putative membrane protein | Membrane protein | 1 |
| [PA14_23920](http://pa14.mgh.harvard.edu/cgi-bin/pa14/view_gene.cgi?GeneID=GID751) | [PA3108](http://pseudomonas.com/AnnotationByPAu.asp?PA=PA3108) | *purF* | Amidophosphoribosyltransferase | Amino acid biosynthesis and metabolism | 1 |
| [PA14_05990](http://pa14.mgh.harvard.edu/cgi-bin/pa14/view_gene.cgi?GeneID=GID962) | [PA0458](http://pseudomonas.com/AnnotationByPAu.asp?PA=PA0458) | *yieO* | Putative drug resistance transporter, emrb/qaca family | Transport of small molecules | 1 |
| [PA14_07720](http://pa14.mgh.harvard.edu/cgi-bin/pa14/view_gene.cgi?GeneID=GID7184) |  |  | Conserved hypothetical protein | Hypothetical protein | 1 |
| [PA14_53670](http://pa14.mgh.harvard.edu/cgi-bin/pa14/view_gene.cgi?GeneID=GID2826) | [PA0820](http://pseudomonas.com/AnnotationByPAu.asp?PA=PA0820) |  | Hypothetical protein | Hypothetical protein | 1 |
| [PA14_37650](http://pa14.mgh.harvard.edu/cgi-bin/pa14/view_gene.cgi?GeneID=GID386) | [PA2077](http://pseudomonas.com/AnnotationByPAu.asp?PA=PA2077) | *odsA* | Conserved hypothetical protein | Hypothetical protein | 0.5 |
| [PA14_72540](http://pa14.mgh.harvard.edu/cgi-bin/pa14/view_gene.cgi?GeneID=GID229) | [PA5497](http://pseudomonas.com/AnnotationByPAu.asp?PA=PA5497) | *nrdJa* | Putative ribonucleotide reductase | Nucleotide biosynthesis and metabolism | 1 |
| [PA14_26880](http://pa14.mgh.harvard.edu/cgi-bin/pa14/view_gene.cgi?GeneID=GID2622) | [PA2877](http://pseudomonas.com/AnnotationByPAu.asp?PA=PA2877) | *bvlR* | Putative transcriptional regulator, lysr family | Transcriptional regulator | 1 |
| [PA14_70100](http://pa14.mgh.harvard.edu/cgi-bin/pa14/view_gene.cgi?GeneID=GID1150) | [PA5309](http://pseudomonas.com/AnnotationByPAu.asp?PA=PA5309) | *pauB4* | Putative Glycine/D-amino acid oxidase | Amino acid biosynthesis and metabolism | 1 |
| [PA14_26110](http://pa14.mgh.harvard.edu/cgi-bin/pa14/view_gene.cgi?GeneID=GID1698) | [PA2933](http://pseudomonas.com/AnnotationByPAu.asp?PA=PA2933) |  | Putative MFS transporter | Transport of small molecules | 1 |
| [PA14_70860](http://pa14.mgh.harvard.edu/cgi-bin/pa14/view_gene.cgi?GeneID=GID2237) | [PA5369](http://pseudomonas.com/AnnotationByPAu.asp?PA=PA5369) | *pstS* | Putative phosphate ABC transporter, periplasmic phosphate-binding protein | Transport of small molecules | 1 |
| [PA14_36330](http://pa14.mgh.harvard.edu/cgi-bin/pa14/view_gene.cgi?GeneID=GID5055) | [PA2193](http://pseudomonas.com/AnnotationByPAu.asp?PA=PA2193) | *hcnA* | Hydrogen cyanide synthase | Central intermediary metabolism | 0.5 |
| [PA14_66310](http://pa14.mgh.harvard.edu/cgi-bin/pa14/view_gene.cgi?GeneID=GID671) | [PA5016](http://pseudomonas.com/AnnotationByPAu.asp?PA=PA5016)(HOMOLOG) | *aceF* | Dihydrolipoamide acetyltransferase | Energy metabolism | 1 |
| [PA14_67220](http://pa14.mgh.harvard.edu/cgi-bin/pa14/view_gene.cgi?GeneID=GID253) | (HOMOLOG) |  | Conserved hypothetical protein | Hypothetical protein | 1 |
| [PA14_65410](http://pa14.mgh.harvard.edu/cgi-bin/pa14/view_gene.cgi?GeneID=GID4039) | [PA4951](http://pseudomonas.com/AnnotationByPAu.asp?PA=PA4951) | *orn* | Oligoribonuclease | Transcription, RNA processing and degradation | 1 |
| [PA14_43950](http://pa14.mgh.harvard.edu/cgi-bin/pa14/view_gene.cgi?GeneID=GID1618) | [PA1588](http://pseudomonas.com/AnnotationByPAu.asp?PA=PA1588) | *sucC* | Succinyl-coa synthetase beta subunit | Energy metabolism | 1 |
| [PA14_67440](http://pa14.mgh.harvard.edu/cgi-bin/pa14/view_gene.cgi?GeneID=GID1057) | [PA5106](http://pseudomonas.com/AnnotationByPAu.asp?PA=PA5106) |  | Putative chlorohydrolase | Putative enzyme | 1 |
| [PA14_53330](http://pa14.mgh.harvard.edu/cgi-bin/pa14/view_gene.cgi?GeneID=GID3207) | [PA0846](http://pseudomonas.com/AnnotationByPAu.asp?PA=PA0846) | *cysZ* | Probable sulfate uptake protein | Transport of small molecules | 0.5 |
| [PA14_10790](http://pa14.mgh.harvard.edu/cgi-bin/pa14/view_gene.cgi?GeneID=GID1496) | [PA4110](http://pseudomonas.com/AnnotationByPAu.asp?PA=PA4110) | *ampC* | Cephalosporinase | Adaptation, protection | 0.5 |
| [PA14_48460](http://pa14.mgh.harvard.edu/cgi-bin/pa14/view_gene.cgi?GeneID=GID4463) |  |  | Probable polyamine binding/transport protein | Transport of small molecules | 1 |
| [PA14_71900](http://pa14.mgh.harvard.edu/cgi-bin/pa14/view_gene.cgi?GeneID=GID5424) | [PA5446](http://pseudomonas.com/AnnotationByPAu.asp?PA=PA5446) |  | Conserved hypothetical protein | Hypothetical protein | 0.5 |
| [PA14_45170](http://pa14.mgh.harvard.edu/cgi-bin/pa14/view_gene.cgi?GeneID=GID1454) | [PA1489](http://pseudomonas.com/AnnotationByPAu.asp?PA=PA1489) |  | Putative oxidoreductase | Putative enzymes | 1 |
| [PA14_29900](http://pa14.mgh.harvard.edu/cgi-bin/pa14/view_gene.cgi?GeneID=GID4407) | [PA2645](http://pseudomonas.com/AnnotationByPAu.asp?PA=PA2645) | *nuoJ* | NADH dehydrogenase I chain J | Energy metabolism | 1 |
| [PA14_01020](http://pa14.mgh.harvard.edu/cgi-bin/pa14/view_gene.cgi?GeneID=GID740) | [PA0084](http://pseudomonas.com/AnnotationByPAu.asp?PA=PA0084) |  | Conserved hypothetical protein | Hypothetical protein | 1 |
| [PA14_07200](http://pa14.mgh.harvard.edu/cgi-bin/pa14/view_gene.cgi?GeneID=GID5405) | [PA0553](http://pseudomonas.com/AnnotationByPAu.asp?PA=PA0553) |  | Hypothetical protein | Hypothetical protein | 1 |
| [PA14_56210](http://pa14.mgh.harvard.edu/cgi-bin/pa14/view_gene.cgi?GeneID=GID3030) | [PA4327](http://pseudomonas.com/AnnotationByPAu.asp?PA=PA4327) |  | Hypothetical protein | Hypothetical protein | 1 |
| [PA14_07660](http://pa14.mgh.harvard.edu/cgi-bin/pa14/view_gene.cgi?GeneID=GID1273) |  |  | Conserved hypothetical protein | Hypothetical protein | 1 |
| [PA14_57600](http://pa14.mgh.harvard.edu/cgi-bin/pa14/view_gene.cgi?GeneID=GID1916) | [PA4434](http://pseudomonas.com/AnnotationByPAu.asp?PA=PA4434) |  | Putative oxidoreductase, aldo/keto reductase family | Energy metabolism | 1 |
| [PA14_01120](http://pa14.mgh.harvard.edu/cgi-bin/pa14/view_gene.cgi?GeneID=GID5394) | PA0092 | *tsi6* | Hypothetical protein | Hypothetical protein | 1 |
| [PA14_50300](http://pa14.mgh.harvard.edu/cgi-bin/pa14/view_gene.cgi?GeneID=GID10) | [PA1091](http://pseudomonas.com/AnnotationByPAu.asp?PA=PA1091) | *fgtA* | Hypothetical protein | Hypothetical protein | 1 |
| [PA14_42050](http://pa14.mgh.harvard.edu/cgi-bin/pa14/view_gene.cgi?GeneID=GID2169) | [PA1739](http://pseudomonas.com/AnnotationByPAu.asp?PA=PA1739) |  | Putative oxidoreductase | Putative enzymes | 1 |
| [PA14_16070](http://pa14.mgh.harvard.edu/cgi-bin/pa14/view_gene.cgi?GeneID=GID1284) | [PA3736](http://pseudomonas.com/AnnotationByPAu.asp?PA=PA3736) | *hom* | Homoserine dehydrogenase | Amino acid biosynthesis and metabolism | 1 |
| [PA14_09990](http://pa14.mgh.harvard.edu/cgi-bin/pa14/view_gene.cgi?GeneID=GID4414) | [PA4166](http://pseudomonas.com/AnnotationByPAu.asp?PA=PA4166) |  | Putative acetyltransferase | Putative enzymes | 1 |
| [PA14_61560](http://pa14.mgh.harvard.edu/cgi-bin/pa14/view_gene.cgi?GeneID=GID1020) | [PA4654](http://pseudomonas.com/AnnotationByPAu.asp?PA=PA4654) |  | Putative MFS transporter | Transport of small molecules | 1 |
| [PA14_68100](http://pa14.mgh.harvard.edu/cgi-bin/pa14/view_gene.cgi?GeneID=GID2273) | [PA5156](http://pseudomonas.com/AnnotationByPAu.asp?PA=PA5156) |  | Conserved hypothetical protein | Hypothetical protein | 1 |
| [PA14_33750](http://pa14.mgh.harvard.edu/cgi-bin/pa14/view_gene.cgi?GeneID=GID1059) | [PA2391](http://pseudomonas.com/AnnotationByPAu.asp?PA=PA2391) | *opmQ* | Putative outer membrane protein precursor | Transport of small molecules | 1 |
| [PA14_16980](http://pa14.mgh.harvard.edu/cgi-bin/pa14/view_gene.cgi?GeneID=GID4858) | [PA3663](http://pseudomonas.com/AnnotationByPAu.asp?PA=PA3663) |  | Conserved hypothetical protein | Hypothetical protein | 1 |
| [PA14_59930](http://pa14.mgh.harvard.edu/cgi-bin/pa14/view_gene.cgi?GeneID=GID4479) |  |  | Conserved hypothetical protein | Hypothetical protein | 1 |
| [PA14_00490](http://pa14.mgh.harvard.edu/cgi-bin/pa14/view_gene.cgi?GeneID=GID556) | [PA0040](http://pseudomonas.com/AnnotationByPAu.asp?PA=PA0040) |  | Putative hemolysin activation/secretion protein | Intracellular trafficking and secretion | 0.5 |
| [PA14_50830](http://pa14.mgh.harvard.edu/cgi-bin/pa14/view_gene.cgi?GeneID=GID225) | [PA1046](http://pseudomonas.com/AnnotationByPAu.asp?PA=PA1046) |  | hypothetical protein | Hypothetical protein | 0.5 |
| [PA14_14690](http://pa14.mgh.harvard.edu/cgi-bin/pa14/view_gene.cgi?GeneID=GID3128) | [PA3817](http://pseudomonas.com/AnnotationByPAu.asp?PA=PA3817) |  | putative rRNA methylase | Translation, post-translational modification, degradation | 1 |
| [PA14_65740](http://pa14.mgh.harvard.edu/cgi-bin/pa14/view_gene.cgi?GeneID=GID392) | [PA4973](http://pseudomonas.com/AnnotationByPAu.asp?PA=PA4973) | *thiC* | thiamin biosynthesis protein ThiC | Biosynthesis of cofactors, prosthetic groups and carriers | 0.5 |
| [PA14_47240](http://pa14.mgh.harvard.edu/cgi-bin/pa14/view_gene.cgi?GeneID=GID3773) | [PA1315](http://pseudomonas.com/AnnotationByPAu.asp?PA=PA1315) |  | putative transcriptional regulator | Transcriptional regulator | 0.5 |

**References**

Jacobs, M. A., Alwood, A., Thaipisuttikul, I., Spencer, D., Haugen, E., Ernst, S., et al. (2003). Comprehensive transposon mutant library of Pseudomonas aeruginosa. *Proceedings of the National Academy of Sciences* 100, 14339–14344. doi: 10.1073/pnas.2036282100.

Létoffé, S., Wu, Y., Darch, S. E., Beloin, C., Whiteley, M., Touqui, L., et al. (2022). Pseudomonas aeruginosa Production of Hydrogen Cyanide Leads to Airborne Control of Staphylococcus aureus Growth in Biofilm and In Vivo Lung Environments. *mBio* 13, e0215422. doi: 10.1128/mbio.02154-22.

Liberati, N. T., Urbach, J. M., Miyata, S., Lee, D. G., Drenkard, E., Wu, G., et al. (2006). An ordered, nonredundant library of Pseudomonas aeruginosa strain PA14 transposon insertion mutants. *Proceedings of the National Academy of Sciences* 103, 2833–2838. doi: 10.1073/pnas.0511100103.

Rahme, L. G., Stevens, E. J., Wolfort, S. F., Shao, J., Tompkins, R. G., and Ausubel, F. M. (1995). Common virulence factors for bacterial pathogenicity in plants and animals. *Science* 268, 1899–1902. doi: 10.1126/science.7604262.

Stover, C. K., Pham, X. Q., Erwin, A. L., Mizoguchi, S. D., Warrener, P., Hickey, M. J., et al. (2000). Complete genome sequence of Pseudomonas aeruginosa PAO1, an opportunistic pathogen. *Nature* 406, 959–964. doi: 10.1038/35023079.
